# Classifying AI-Powered prediction models for disability progression using the Tamir-Based complex fuzzy Aczel–Alsina WASPAS method

**DOI:** 10.1038/s41598-025-12296-w

**Published:** 2025-08-13

**Authors:** Jabbar Ahmmad, Meraj Ali Khan, Ibrahim Aldayel, Tahir Mahmood

**Affiliations:** 1 Department of Mathematics and Statistics, International Islamic University Islamabad, Islamabad, Pakistan; 2 Department of Mathematics and Statistics, College of Science, Imam Mohammad Ibn Saud Islamic University (IMSIU), Riyadh, 11566 Saudi Arabia; 3 King Salman Center for Disability Research, Riyadh, 11614 Saudi Arabia

**Keywords:** Disability conditions, AI-powered models, Aczel-Alsina t-norm and t-conorm, Complex fuzzy set, WASPAS approach, Health care, Mathematics and computing

## Abstract

Tracking the development of disability conditions presents significant challenges due to uncertainty, imprecision, and dynamic health progression patterns. Traditional multi-criteria decision-making (MCDM) techniques often struggle with such complex and fuzzy medical data. To address this gap, we propose a novel classification framework based on Tamir’s complex fuzzy Aczel-Alsina weighted aggregated sum product assessment (WASPAS) approach. This hybrid model incorporates complex fuzzy logic to handle multidimensional uncertainty and utilizes the Aczel-Alsina function for flexible aggregation. We apply this method to evaluate and classify AI-powered predictive models used for monitoring disability progression. The proposed framework not only improves classification accuracy but also enhances decision support in healthcare planning. A case study validates the robustness, sensitivity, and effectiveness of the proposed method in real-world disability tracking scenarios.

## Introduction

Prediction models driven by artificial intelligence (AI) are incredibly complex computer programs that foresee future occurrences using historical and real-time data. Several important machine learning techniques are used to help identify complex patterns and correlations in the data, including regression, neural networks, and ensemble approaches. Unlike the traditional models, AI-powered approaches learn and improve over time with continuous learning. Some of the key applications include weather forecasting, financial markets analysis, diagnostics of healthcare, as well as demand forecasting within the supply chain. The structured and unstructured data will be analyzed by these models, generating extremely accurate and reliable forecasts. Deep learning within the field of machine learning completely shifted the dynamics of prediction models because it was capable of handling large data sets and many operations, such as image recognition and natural language processing. Integration with big data frameworks through AI is making it even more possible to make real-time predictions in dynamic systems. These models make insights actionable, minimize uncertainty, and optimize resource allocation, all improving decision-making. They predict disease outbreaks in health, for example, or network congestion in telecommunications. The range and the efficiency of these models increase as AI technologies evolve and thus impacting and changing the fate of industries and improving the lives of individuals worldwide.

**Q. Why AI-Powered Prediction Models for Tracking the Development of Disability Conditions?**

Since AI-based predictive models are efficient in handling large and complex data sets, they are crucial in tracking the arising handicap problems. They apply the most advanced algorithms that allow them to identify risk factors correctly and detect trends, and predict the course of a disease’s development. They can present personalized insights for improving early diagnosis and intervention by bringing together clinical data, the history of the patient, and current monitoring. These predictive capabilities allow medical professionals to design specific rehabilitative programs that result in maximum benefits for patients with a minimum employment of resources. AI models can also learn continuously and improve their predictions based on new data assimilation. Their scalability and velocity make them a good choice for managing populations, hence enabling proactive and cost-effective healthcare strategies. This brings about an improved quality of life for individuals and reduces long-term societal burdens of disability.

**Q. What is the importance of AI-powered models in analyzing the progression of Human disability conditions?**

The importance of AI-powered models in analyzing the progression of human disability conditions is given in the following Table 1.

**Table 1:** Importance of AI-powered models in human disability.

Aspects	Importance
Personalized insight	To offer individualized therapy and management advice, they examine the data of each patient.
Predictive analysis	AI assists clinicians in proactively planning interventions by forecasting the future course of a condition.
Data Integration	Integrates information from several sources (such as sensors, clinical records, and medical imaging) to provide a comprehensive analysis.
Rehabilitation Guidance	Offers suggestions on the best rehabilitation methods for particular ailments.
Patient empowerment	AI-powered solutions provide patients with useful information, encouraging self-management and active involvement in their care.

Different researchers have produced their work in the AI field regarding human disability. We can see from the literature that Aruna et al.^1^ have discovered AI-powered real-time communications systems for people with disability. Moreover, Olawade et al.^2^ discovered the role of AI in enhancing health care for people with disabilities. Brotosaputro et al.^3^ explored AI-powered assistive technologies for improved accessibility. Chopra et al.^4^ proposed the utilization of AI and innovative tech stacks to support students with learning disabilities in higher education. Habbal et al.^5^ studied human disability and empowered assistive technologies for people with disabilities to improve the lifestyle of the disabled. Shuford et al.^6^ explored the contribution of AI in improving accessibility for individuals with disabilities.

## Literature review

The idea of fuzzy set was introduced by Zadeh^7^ as a generalization of crisp set theory. In the case of crisp set theory, the membership function is defined from any non-empty universal set to {0, 1} while in the case of fuzzy set, the membership function is defined from any non-empty universal set to [0, 1]. FS has many applications in different fields, like Zimmermann et al.^8^ developed the application of FS in mathematical programming. Wong and Lai^9^ produced and developed a survey of FS theory in production and management. Liou et al.^10^ used the FS in environmental engineering and discussed the application of FS in the evaluation of the river quality in Taiwan. McBratney and Moore^11^ explored the application of FS to climate change. Mardani et al.^12^ introduced a decision-making algorithm based on FS and utilized the proposed notions in healthcare and medical problems. Dereli et al.^13^ developed the industrial application of type-2 FS and systems. Moreover, Gen et al.^14^ introduced the application of FS theory in inventory control models. FS has its application in human disability, and many developments have been made in this regard. Costa et al.^15^ proposed FS FS-based model that helps in the perception of disability from a public health perspective. Hezam et al.^16^ utilized the fermatean fuzzy double normalization-based multiple aggregation methods in assessing barriers to sustainable transportation systems for persons with disabilities. Manghirmalani et al.^17^ discussed the fuzzy approach to classifying learning disability. Hossain et al.^18^ explored the theoretical and practical investigation of the fuzzy AHP approach to design communication systems for disabled individuals.

The notion of FS was further generalized into two different forms of the complex fuzzy set (CFS), one was developed by Ramot et al.^19^ and the other was produced by Tamir et al.^20^. Although both structures are independent, we can observe that the Tamir et al.^20^ approach is flexible and easy to use. In Ramot et al.^19^ structure the membership grade is categorized by amplitude and phase term, and there is a condition that the phase terms $\:"r"$ must belong to [0, 1]. But in a situation where the data is of the form $\:0.7+\iota\:0.6$ then we can observe that the amplitude term $\:r=\sqrt{{0.7}^{2}+{0.8}^{2}}\notin\:\left[0,\:1\right]$ and in this case, only the Tamir et al.^20^ approach can be feasible and handy to tackle such a kind of complex fuzzy data. The application of both these structures is discussed by the researchers. Tamir et al.^20^ proposed an overview and application of CFS. CFS has an application in signals as discussed by Ma et al.^21^. Chen et al.^22^ utilized CFS in the neurofuzzy architecture. Hu et al.^23^ explored the orthogonality between CFS and proposed its application in signal detection. Li et al.^24^ explored the self-learning complex neuro-fuzzy systems with CFS and developed their applications for adaptive image noise canceling. Also, Ma et al.^25^ developed a method for multiple periodic factor prediction problems with the aid of CFS. Hu et al.^26^ explored the distance and continuity of CFS in their research. More literature on neutrosophic set, interval valued Fermatean Fuzzy set, interval values spherical fuzzy sets and intuitionistic fuzzy set, and q-rung orthopair fuzzy set is given in the form of Table 2.

**Table 2:** Description of the literature.

Different Approaches	Methods used	Structure	Applications
Banik et al^27^. approach	Extended COPRAS Technique	Neutrosophic Sets	Highway Project Planning Oriented MCGDM Problem
Chatterjee et al^28^. approach	MCGDM Approach	Neutrosophic Sets	To Detect the Best Solar Panel Installation Provider
Haque et al^29^. approach	MCGDM	Neutrosophic Sets	E-learning app selection
Seikh and Mandal^30^ approach	PROMETHEE II Method	Interval Valued Fermatean Fuzzy set	Bio-medical waste management
Mandal and Seikh^31^ approach	MABAC technique	Interval-valued spherical fuzzy set	Selection of the Plastic waste management process
Seikh and Chatterji^32^ approach	MADM Approach	Intuitionistic fuzzy set	Evaluation and Selection of an E-learning Website
Seikh and Mandal^33^ approach	MADM Approach	q-rung orthopair fuzzy set	Cite selection for software operating units

### Motivation of the proposed and key novelties

The development of disability conditions tends to be non-linear, indeterminate, and subject to various medical, environmental, and behavioral factors. As the use of artificial intelligence (AI) increases in the healthcare sector, a number of forecast models have been introduced to predict disability development. The choice of the most appropriate AI model for a given context, however, continues to be a difficult task owing to the natural vagueness and multidimensionality of medical data. Conventional decision-making methods, such as fuzzy sets, tend to be ineffective in supporting such complexities, resulting in inferior model classification and decision assistance. This work is driven by the necessity for a stronger, uncertainty-aware decision mechanism to better assess and classify AI-driven predictive models with better accuracy and explainability. Specifically, current MCDM techniques fail to leverage the full potential of complex fuzzy logic when combined with sophisticated aggregation operators developed with deep uncertainty in mind.

The major Novelties of this research are as follows:

- Innovation of a novel hybrid MCDM system that combines Tamir’s complex fuzzy sets with the Aczel-Alsina function within the WASPAS framework to facilitate accurate management of uncertainty and interdependence in medical information.
- Creation of a new mechanism for classifying AI models that are aimed at monitoring the progression of disability, central to proactive intervention and rehabilitation planning.
- Real-world application of the suggested method, its testing for performance via sensitivity analysis, and comparative performance metrics.
- Through a detailed identification of the deficiencies in existing approaches and formulating a comprehensive framework, the study leaves a deep mark on fuzzy decision-making, healthcare informatics, and AI-based medical model evaluation.

### Research gaps and contributions

Despite the rapid advancement of AI technologies in healthcare, the accurate classification and selection of AI-based prediction models for disability tracking remain underexplored. Most existing approaches fail to handle the complex uncertainty embedded in disability-related datasets and ignore interrelated criteria during model evaluation. Traditional MCDM techniques like TOPSIS, VIKOR, or AHP are not well-suited for processing fuzzy, imprecise, or hesitant information, which is often critical in medical decision-making contexts.

A detailed review of the literature shows the following research gaps:

1. Existing works inadequately address complex fuzzy uncertainty in evaluating predictive models for chronic or progressive disability conditions.
2. There is a lack of hybrid MCDM frameworks that can combine mathematical robustness with medical interpretability.
3. Very few studies have applied Aczel-Alsina aggregation within the context of complex fuzzy sets for healthcare decision-making.
4. Sensitivity and stability analyses of AI-based decision models under complex fuzzy environments are rarely conducted or systematically reported.

To fill these gaps, this study formulates the following research questions:

- How can the evaluation and classification of AI-powered prediction models be improved under uncertain, imprecise, and interdependent medical data conditions?
- Can the integration of Tamir’s complex fuzzy sets with Aczel-Alsina aggregation enhance the robustness and interpretability of MCDM methods in healthcare?
- What is the comparative performance of the proposed framework against traditional MCDM techniques in terms of ranking stability and decision consistency?

To address these questions, the following key contributions are made:

- A novel Tamir’s Complex Fuzzy Aczel-Alsina WASPAS model is proposed to classify AI prediction models in uncertain medical environments.
- The framework introduces a new aggregation mechanism that captures both the fuzziness and interrelation of evaluation criteria.
- A comparative and sensitivity analysis validates the performance and robustness of the proposed approach.
- The study enhances practical decision-making in healthcare by offering an interpretable, flexible, and stable evaluation tool for disability prediction models.

### Study framework

The arrangement of the article is as follows: In Sect. 2, we have proposed a literature review of the existing notions. We have also discussed the problem statement and the contribution of the developed approach in this section. In Sect. 3, we have delivered some basic definition and their fundamental laws. Section 4 is about the Aczel-Alsina operating rules for Tamir’s CFNs. In Sect. 5, we have developed the aggregation theory of complex fuzzy Aczel-Alsina (CFAA) aggregation operators (AOs). In Sect. 6, we have proposed the WASPAS approach for the developed aggregation theory, and we have also discussed the application of these developed approaches to classify the AI-powered prediction models to track the progression of human disability. Section 7 is about the comparative analysis of the delivered approach to discuss the advantages of the initiated work. Moreover, Sect. 8 is about the stability analysis of the initiated theory to discuss the stability of developed AOs. In Sect. 9, we have discussed the conclusion of the developed work.

**Fig. 1:**
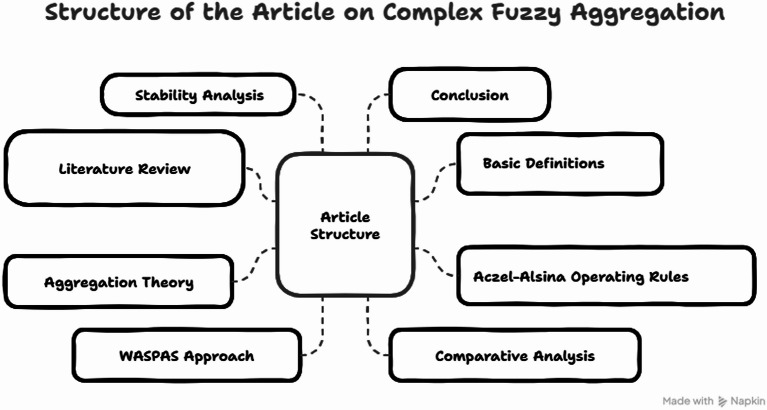
Flow chart of the proposed study.

Moreover, the flow chart of the proposed methodology is given in Fig. 1.

## Preliminaries

This section of the article is to define the fundamental notions that are used in our further study. The notions of Ramot CFS and Tamir’s CFS are defined to distinguish between these two ideas.

### Definition 1

^19^ Let $\:X$ be a non-empty universal set. The Ramot’s structure of CFS is given by

$$\:\mathfrak{I}=\left\{x,\:f\left(x\right)|f\left(x\right)=r\left(x\right){e}^{\iota\:\omega\:\left(x\right)}\right\}$$

Where $\:r\left(x\right)$ is the amplitude terms and $\:\omega\:\left(x\right)$ is the phase term.

### Definition 2

^20^ Let $\:X$ be a non-empty universal set and $\:f:X\to\:\left[0,\:1\right]$ such that

$$\:\mathfrak{I}=\left\{x,\:f\left(x\right)|f\left(x\right)=a\left(x\right)+\iota\:b\left(x\right)\:for\:all\:x\in\:X\right\}$$

is called Tamir’s CFS. Where $\:f\left(x\right)=a\left(x\right)+\iota\:b\left(x\right)$ is the membership function and $\:a\left(x\right),\:b\left(x\right)\in\:\left[0,\:1\right]$ and called the real and imaginary parts of membership grades. Note that all $\:a\left(x\right)+\iota\:b\left(x\right)$ belong to unit square provides the flexibility of handling complex fuzzy information. For the sake of simplicity, $\:\mathfrak{I}=\left\{a+\iota\:b\right\}$ is called CFN.

## Aczel-Alsina operations of CFNs

We have presented the theory of complex fuzzy Aczel-Alsina operational laws in this section, which is based on the theory of Aczel-Alsina t-norm and t-conorm. Additionally, we have demonstrated a few basic features based on these operations. The general conversation is provided as.

### Definition 3

Assume that $\:{\mathfrak{I}}_{1}=\left\{{{a}}_{1}+\iota{b}_{1}\right\}$ and $\:{\mathfrak{I}}_{2}=\left\{{{a}}_{2}+\iota{b}_{2}\right\}$are two CFNs with $\:\ell>0$ and $\:\mathcal{r}\ge\:1$, then the fundamental operating rules for CFNs based on Aczel-Alsina t-norm and t-conorm are given by

- $$\:{\mathfrak{I}}_{1}\otimes\:{\mathfrak{I}}_{2}=\left({{e}^{-\left({\left(-\text{log}\left({{a}}_{1}\right)\right)}^{\mathcal{r}}+{\left(-\text{log}\left({{a}}_{2}\right)\right)}^{\mathcal{r}}\right)}}^{\frac{1}{\mathcal{r}}}+\iota\:\:\left({{e}^{-\left({\left(-\text{log}\left({b}_{1}\right)\right)}^{\mathcal{r}}+{\left(-\text{log}\left({b}_{2}\right)\right)}^{\mathcal{r}}\right)}}^{\frac{1}{\mathcal{r}}}\right)\right)$$ 
- $$\:{\mathfrak{I}}_{1}\otimes\:{\mathfrak{I}}_{2}=\left({{e}^{-\left({\left(-\text{log}\left({{a}}_{1}\right)\right)}^{\mathcal{r}}+{\left(-\text{log}\left({{a}}_{2}\right)\right)}^{\mathcal{r}}\right)}}^{\frac{1}{\mathcal{r}}}+\iota\:\:\left({{e}^{-\left({\left(-\text{log}\left({b}_{1}\right)\right)}^{\mathcal{r}}+{\left(-\text{log}\left({b}_{2}\right)\right)}^{\mathcal{r}}\right)}}^{\frac{1}{\mathcal{r}}}\right)\right)$$ 
- $$\:\ell\,{\mathfrak{I}}_{1}=\left(1-{e}^{-{\left({\ell\,\left(-\text{log}\left(1-{a}_{1}\right)\right)}^{\ell\,{r}}\right)}^{\frac{1}{\mathcal{r}}}}+\iota\:\:\left(1-{e}^{-{\left({\ell\,\left(-\text{log}\left(1-{b}_{1}\right)\right)}^{\mathcal{r}}\right)}^{\frac{1}{\mathcal{r}}}}\right)\right)\:$$ 
- $$\:{\mathfrak{I}}_{1}^{\ell}=\left(1-{e}^{-{\left({\ell\left(-\text{log}\left(1-{a}_{1}\right)\right)}^{\mathcal{r}}\right)}^{\frac{1}{\mathcal{r}}}}+\iota\:\:\left(1-{e}^{-{\left({\ell\left(-\text{log}\left(1-{b}_{1}\right)\right)}^{\mathcal{r}}\right)}^{\frac{1}{\mathcal{r}}}}\right)\right)\:$$

### Definition 4

For ranking the two CFNs, the notion of score function and accuracy function helps in this regard. For a CFN $\:\mathfrak{I}=\left\{a+\iota\:b\right\}.$ The idea of the score function and accuracy function is given by

$$\:Scr.\left(\mathfrak{I}\right)=\frac{1}{2}\left(a+b\right)\in\:\left[0,\:1\right]$$

And

$$\:Acur.\:\left(\mathfrak{I}\right)=\frac{1}{2}\left(a-b\right)\in\:\left[-1,\:1\right]$$

### Theorem 1

Assume that $\:{\mathfrak{I}}_{1}=\left\{\left({{a}}_{1}+\iota\:{b}_{1}\right)\right\}$ and $\:{\mathfrak{I}}_{2}=\left\{\left({{a}}_{2}+\iota\:{b}_{2}\right)\right\}\:$denote two CFNs with $\:\ell,\ell\,,{\ell}_{1},\:{\ell}_{2}>0$. Then we have the following properties

- $$\:{\mathfrak{I}}_{1}\oplus\:{\mathfrak{I}}_{2}={\mathfrak{I}}_{2}\oplus\:{\mathfrak{I}}_{1}$$ 
- $$\:{\mathfrak{I}}_{1}\otimes\:{\mathfrak{I}}_{2}={\mathfrak{I}}_{2}\otimes\:{\mathfrak{I}}_{1}$$ 
- $$\:\ell\left({\mathfrak{I}}_{1}\oplus\:{\mathfrak{I}}_{2}\right)={\ell\mathfrak{I}}_{1}\oplus\:{\ell\mathfrak{I}}_{2}$$ 
- $$\:\left({\ell}_{1}+{\ell}_{2}\right){\mathfrak{I}}_{1}={\ell}_{1}{\mathfrak{I}}_{1}+{{\ell}_{2}\mathfrak{I}}_{1}$$ 
- $$\:{\mathfrak{I}}_{1}^{{\ell}_{1}}\otimes\:{\mathfrak{I}}_{1}^{{\ell}_{2}}={\mathfrak{I}}_{1}^{\left({\ell}_{1}+{\ell}_{2}\right)\:}$$ 
- $$\:{\mathfrak{I}}_{1}^{{\ell}_{1}}\otimes\:{\mathfrak{I}}_{1}^{{\ell}_{2}}={\mathfrak{I}}_{1}^{\left({\ell}_{1}+{\ell}_{2}\right)\:}$$

## Aczel-Alsina aggregation operators using tamir’s CF information

This section of the article is about the aggregation theory for Tamir’s CFNs. We have delivered the idea of CFAAWA and CFAAWG AOs.

Here in this section $\:{\mathfrak{I}}_{\mathcal{j}}=\left\{{{a}}_{\mathcal{j}}+\iota\:{b}_{\mathcal{j}}\right\}$ $\:\left(\,\,\mathcal{j}=1,\:2,\:3,\dots\:,\:\mathbbm{n}\right)$ denote the collection of CFNs and $\:\mathcal{w}=\left({\mathcal{w}}_{1},\:{\mathcal{w}}_{2},\dots\:,\:{\mathcal{w}}_{\mathbbm{n}}\right)$ denote weight vectors (WVs) for $\:{\mathfrak{I}}_{\mathcal{j}}\left(\,\,\mathcal{j}\,=1,\:2,..,\:\mathbbm{n}\right)$ with $\:{\mathcal{w}}_{\mathcal{j}}\in\:\left[0,\:1\right]$ and $\:\sum\:_{\mathcal{j}=1}^{\mathbbm{n}}{\mathcal{w}}_{\mathcal{j}}=1$.

### Definition 5

The idea of CFAAWA AO is of the form

$$\:CFAAW{A}_{\mathcal{w}}\left({\mathfrak{I}}_{1},\:{\mathfrak{I}}_{2},\dots\:,\:{\mathfrak{I}}_{\mathbbm{n}}\right)=\begin{array}{c}\mathbbm{n}\\\:\oplus\:\\\:\mathcal{j}=1\end{array}\left({\mathcal{w}}_{\mathcal{j}}{\mathfrak{I}}_{\mathcal{j}}\right)$$

$$\:=\left(1-{e}^{-{\left(\sum\:_{\mathcal{j}\,=1}^{\mathbbm{n}}{\mathcal{w}}_{\mathcal{j}}{\left(-\text{log}\left(1-{\mathcal{a}}_{\mathcal{j}}\right)\right)}^{\mathcal{r}}\right)}^{\frac{1}{\mathcal{r}}}}+\:\iota\:\:\left(1-{e}^{-{\left(\sum\:_{\mathcal{j}\,=1}^{\mathbbm{n}}{\mathcal{w}}_{\mathcal{j}}{\left(-\text{log}\left(1-{b}_{\mathcal{j}}\right)\right)}^{\mathcal{r}}\right)}^{\frac{1}{\mathcal{r}}}}\right)\right)\:$$

### Definition 6

The idea of CFAAWG AO is of the form

$$\:CFAAW{G}_{\mathcal{w}}\left({\mathfrak{I}}_{1},\:{\mathfrak{I}}_{2},\dots\:,\:{\mathfrak{I}}_{\mathbbm{n}}\right)=\begin{array}{c}\mathbbm{n}\\\:\otimes\\\:\mathcal{j}=1\end{array}{\left({\mathfrak{I}}_{\mathcal{j}}\right)}^{{\mathcal{w}}_{\mathcal{j}}}$$

$$\:=\left({e}^{-{\left(\sum\:_{\mathcal{j}\,=1}^{\mathbbm{n}}{\mathcal{w}}_{\mathcal{j}}{\left(-\text{log}\left({\mathcal{a}}_{\mathcal{j}}\right)\right)}^{\mathcal{r}}\right)}^{\frac{1}{\mathcal{r}}}}+\:\iota\:\:\left({e}^{-{\left(\sum\:_{\mathcal{j}\,=1}^{\mathbbm{n}}{\mathcal{w}}_{\mathcal{j}}{\left(-\text{log}\left({b}_{\mathcal{j}}\right)\right)}^{\mathcal{r}}\right)}^{\frac{1}{\mathcal{r}}}}\right)\right)$$

## Application of the proposed theory

In this part of the article, we have discussed a decision-making approach to show the utilization of the delivered work. We have defined an algorithm for the WASPAS technique under the notion of CFAAWA AOs and CFAAWG AOs. For this purpose, assume that there are $\:\mathbbm{m}$ alternatives and $\:{\mathcal{T}}_{alt.}=\left\{{\mathcal{T}}_{alt-1},\:{\mathcal{T}}_{alt-2},\dots\:,\:{\mathcal{T}}_{alt-\mathbbm{m}}\right\}$ represents the set of $\:"\mathbbm{m}"$ alternatives. Also, the set of $\:"\mathbbm{n}"$ attributes is given as $\:{\mathcal{S}}_{atr.}=\left\{{\mathcal{S}}_{atr.-1},\:{\mathcal{S}}_{atr.-2},\:\dots\:,\:{\mathcal{S}}_{atr.-\mathbbm{n}}\right\}.$ The WVs of these attributes are $\:{\mathcal{w}}_{\mathcal{j}}={\left({\mathcal{w}}_{1},{\mathcal{w}}_{2},\dots\:,{\mathcal{w}}_{\mathbbm{n}}\right)}^{T}$ such that $\:\sum\:_{\mathcal{j}\,=1}^{\mathbbm{n}}{\mathcal{w}}_{\mathcal{j}}=1$ and $\:{\mathcal{w}}_{\mathcal{j}}\in\:\left[0,\:1\right]\mathcal{\:}\forall\:\mathcal{\:}\mathcal{j}$. Assume that the experts provide their assessment in Tamir’s CFNs given by $\:{\mathfrak{I}}_{\mathbbm{i}\mathcal{j}}\,=\left\{{\mathcal{a}}_{\mathbbm{i}\mathcal{j}}+\iota\:{b}_{\mathbbm{i}\mathcal{j}}\right\}$ for $\:\mathbbm{i}=1,\:\text{2,3},\:..,\:\mathbbm{m}$ and $\:\mathcal{j}=1,\:2,\:3,\:\dots\:,\:\mathbbm{n}.$

The proposed WASPAS algorithm is given by.

### The WASPAS algorithm

#### Step 1

The data provided by the experts is collected in the form of a matrix consisting of CFNs.

$$\:\mathbbm{m}=\left[\begin{array}{ccc}{\mathfrak{I}}_{11}&\:{\mathfrak{I}}_{12}&\:{\mathfrak{I}}_{1\mathcal{j}}\\\:{\mathfrak{I}}_{21}&\:{\mathfrak{I}}_{22}&\:{\mathfrak{I}}_{2\mathcal{j}}\\\:\begin{array}{c}\cdots\\\:{\mathfrak{I}}_{\mathbbm{i}1}\end{array}&\:\begin{array}{c}\ddots\:\\\:{\mathfrak{I}}_{\mathbbm{i}2}\end{array}&\:\begin{array}{c}\cdots\\\:{\mathfrak{I}}_{\mathbbm{i}\mathcal{j}}\end{array}\end{array}\right]$$

**Step 2**:

#### Case I

If attributes are benefit type, then use the following formula.

$$\:{\mathfrak{I}}_{\mathbbm{i}\mathcal{j}}^{*}={\mathfrak{I}}_{\mathbbm{i}\mathcal{j}}$$

It means no change in the data obtained from step 1.

#### Case-II

If the attributes are cost type, then take the complement of CFN, and its formula is given by.

$$\:{\mathfrak{I}}_{\mathbbm{i}\mathcal{j}}^{*}={\left({\mathfrak{I}}_{\mathbbm{i}\mathcal{j}}\right)}^{c}\:\text{w}\text{h}\text{e}\text{r}\text{e}\:{\left({\mathfrak{I}}_{\mathbbm{i}\mathcal{j}}\right)}^{c}=\left\{{1-\mathcal{a}}_{\mathbbm{i}\mathcal{j}}+\iota\:{1-b}_{\mathbbm{i}\mathcal{j}}\right\}.$$

Hence, the collective formulation is given in the following formula

$$\:\mathbbm{n}=\left\{\begin{array}{cc}{\mathfrak{I}}_{\mathbbm{i}\mathcal{j}}&\:\text{f}\text{o}\text{r}\:\text{b}\text{e}\text{n}\text{e}\text{f}\text{i}\text{t}\:\text{t}\text{y}\text{p}\text{e}\:\text{a}\text{t}\text{t}\text{r}\text{i}\text{b}\text{u}\text{t}\text{e}\text{s}\\\:{\left({\mathfrak{I}}_{\mathbbm{i}\mathcal{j}}\right)}^{c}&\:\text{f}\text{o}\text{r}\:\text{c}\text{o}\text{s}\text{t}-\text{t}\text{y}\text{p}\text{e}\:\text{a}\text{t}\text{t}\text{r}\text{i}\text{b}\text{u}\text{t}\text{e}\text{s}\end{array}\right.$$

Where $\:{\left({\mathfrak{I}}_{\mathbbm{i}\mathcal{j}}\right)}^{c}=\left\{\left(\left(1-\overline{{\mathcal{a}}_{\mathbbm{i}\mathcal{j}}}\right)+\iota\:\left(1-\overline{{b}_{\mathbbm{i}\mathcal{j}}}\right),\:\left(1-\underset{\_}{{a}_{\mathbbm{i}\mathcal{j}}}\right)+\iota\:\left(1-\underset{\_}{{b}_{\mathbbm{i}\mathcal{j}}}\right)\right)\right\}$ represent the complement of $\:{\mathfrak{I}}_{\mathbbm{i}\mathcal{j}}.$

#### Step 3

The relative importance of the alternative is denoted and computed by using the formula of CFAAWA AO, that is.

$$\:{\mathcal{H}}_{\mathcal{j}}^{WS}=\left(1-{e}^{-{\left(\sum\:_{\mathcal{j}\,=1}^{\mathbbm{n}}{\mathcal{w}}_{\mathcal{j}}{\left(-\text{log}\left(1-{\mathcal{a}}_{\mathcal{j}}\right)\right)}^{\mathcal{r}}\right)}^{\frac{1}{\mathcal{r}}}}+\:\iota\:\:\left(1-{e}^{-{\left(\sum\:_{\mathcal{j}\,=1}^{\mathbbm{n}}{\mathcal{w}}_{\mathcal{j}}{\left(-\text{log}\left(1-{b}_{\mathcal{j}}\right)\right)}^{\mathcal{r}}\right)}^{\frac{1}{\mathcal{r}}}}\right)\right)$$

#### Step 4

Similarly, find out $\:{\mathcal{H}}_{\mathcal{j}}^{WP}$ by using the formula

$$\:{\mathcal{H}}_{\mathcal{j}}^{WP}=\left({e}^{-{\left(\sum\:_{\mathcal{j}\,=1}^{\mathbbm{n}}{\mathcal{w}}_{\mathcal{j}}{\left(-\text{log}\left({\mathcal{a}}_{\mathcal{j}}\right)\right)}^{\mathcal{r}}\right)}^{\frac{1}{\mathcal{r}}}}+\:\iota\:\:\left({e}^{-{\left(\sum\:_{\mathcal{j}\,=1}^{\mathbbm{n}}{\mathcal{w}}_{\mathcal{j}}{\left(-\text{log}\left({b}_{\mathcal{j}}\right)\right)}^{\mathcal{r}}\right)}^{\frac{1}{\mathcal{r}}}}\right)\right)$$

#### Step 5

Finally, utilize the WASPAS technique for alternative by.

$$\:{\mathcal{H}}_{\mathcal{j}}=\frac{{\mathcal{H}}_{\mathcal{j}}^{WS}+{\mathcal{H}}_{\mathcal{j}}^{WP}}{2}$$

#### Step 6

Now, find out the score values of the results obtained from Step 5 and rank the alternatives according to the results.

**Fig. 2:**
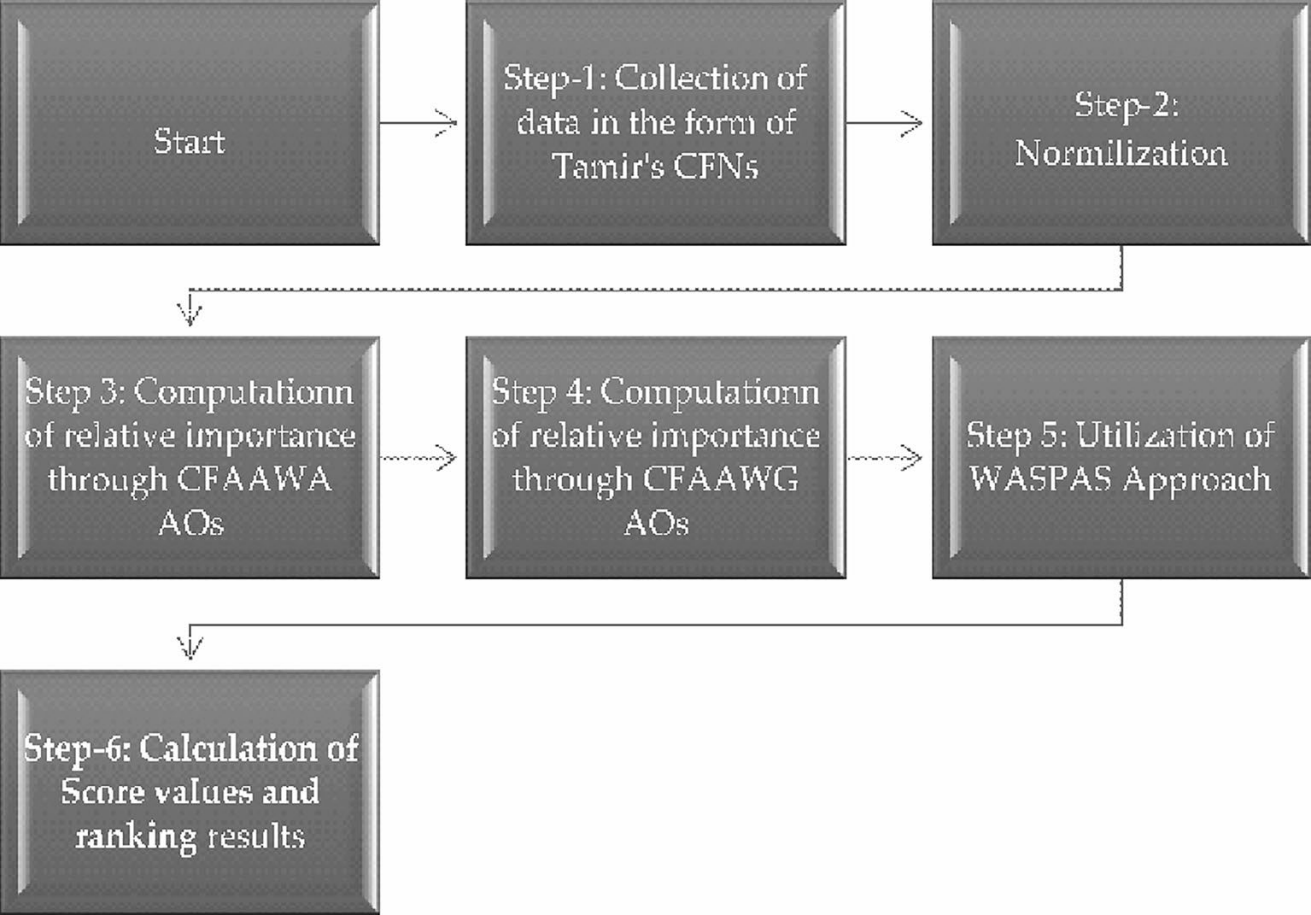
Flow chart of the proposed algorithm for the WASPAS approach.

The flow chart of the proposed algorithm is given in Fig. 2.

### Illustrated example

AI-based predictive models change the health environment through unique capabilities to monitor and understand the genesis of handicapped illnesses. These models track patterns from large databases of clinical records, imaging, genetic data, and real-time patient data that could predict the trajectory of impairment. These models will enable state-of-the-art neural networks and machine learning techniques so that complex nonlinear correlations among the factors predicting the disease course can be identified. AI enables early intervention in the avoidance or reduction of disability and enhances early detection based on even the slightest indications of functional decline. It not only gives predictions for every patient profile but also about the lifestyle, surroundings, and genetic predispositions. AI further helps in designing rehabilitation plans for adaptive therapy so that they keep updating because of the patient’s development. Natural language processing further expands its analysis of the patient’s response through a better understanding of subjective feelings of handicap. It supports the massive epidemiological research study in AI models that expose social trends and allow policymakers to gain insight into the issues. Merging wearable technology with telehealth systems allows for democratized access to advanced predictive technologies for patients and clinicians. Data privacy, biased algorithms, and transparency are ongoing concerns. Overall, applying AI-powered prediction models to precision medicine will revolutionize precision medicine and improve the outcomes for patients. Algorithmic bias remains, along with data privacy and transparency. All these factors will then ensure that prediction models, powered by AI, definitely revolutionize precision medicine and patient care.

AI-powered prediction models are given by.

#### Logistic regression (LR)

Logistic regression is a fundamental model for binary classification that forecasts the likelihood of acquiring a handicap condition. It looks at how lifestyle factors, including age and exercise levels, relate to medical conditions. Because of its simplicity, healthcare applications can easily interpret it. Regularization techniques also deal with overfitting in complex datasets. It assumes linear reparability and is not effective for more complex patterns.

#### Support vector machines (SVMs)

SVMs classify data by finding the most suitable hyperplanes in a hyperspace. For disability prediction, it distinguishes between patients and various risks using medical data. The kernel trick allows capturing the nonlinear relationships very appropriately. SVMs are accurate when the datasets are small, since accuracy and reliability have to be ensured. Still, they are computationally expensive when the dataset is larger.

#### Gradient boosting machines (GBMs)

GBMs construct trees iteratively, correcting the mistakes that the previous models made to improve predictions. In disability studies, they fine-tune insights on slow-developing conditions such as arthritis or muscular degeneration. The model is scalable and will perform well on large health datasets. Fine-tuning hyperparameters boosts prediction precision. However, they require a lot of computational resources.

#### Random forest (RF)

A random forest ensemble learning model makes multiple decision trees and averages their predictions for greater accuracy. In tracking disabilities, it analyzes diverse health factors, including diet, exercise, and comorbidities, in determining risk. It also manages missing data well and highlights important predictors. Random forests can deal with high-dimensional datasets. Its interpretability is a little challenging compared to simpler models.

#### Neural network (NN)

Neural networks are strong discovery instruments for discovering patterns in huge, complex data. For disability, they process unstructured data such as images, gait patterns, or EEG signals. It has layers of connected nodes, emulating brain activity and abstracting deep insights from raw data. The methodology requires less manual involvement to learn various deficiencies. However, they are more difficult to comprehend and require large amounts of data.

#### Convolutional neural network (CNN)

CNNs specialize in image and spatial data processing. In the area of disability diagnostics, they are the best at processing MRI or CT scans to detect conditions such as cerebral palsy or spinal cord injuries. They can automatically recognize complex patterns in medical images through hierarchical feature extraction. Transfer learning increases efficiency if datasets are small. Nevertheless, CNNs suffer from difficulties in generalizing data.

#### Recurrent neural network (RNN)

RNNs are best suited for processing sequential data, such as time-series data, like patient health trends or speech impairments. They track the progression of disabilities by analyzing past data to predict future conditions. Long Short-Term Memory (LSTM) units address long-range dependencies in datasets. These models are good at detecting recurring patterns over time. However, they are computationally intensive.

#### Neural Language processing (BLP)

NLP models parse text data, for instance, clinical notes, to discover the early symptoms of disability in unstructured records. With patient feedback, sentiment analysis keeps track of emotional and psychological disabilities. Contextualized language models like BERT or GPT provide contextual awareness. They automatically extract large health datasets. However, to make this accurate, domain-specific adaptation is necessary.

#### K-nearest neighbors (KNN)

KNN is an easy algorithm that classifies data based on proximity to its nearest neighbors. It predicts the risk of disability based on a comparison of patients with similar medical profiles. KNN is non-parametric and, hence, flexible for different datasets. It is easy to implement for small-scale studies of disability. It becomes computationally expensive when the dataset is large.

#### Bayesian network (BN)

BN uses probabilistic inference to model dependencies between variables. They are particularly effective for understanding the interplay of genetic, environmental, and lifestyle factors in disability onset. The model updates predictions dynamically as new evidence becomes available. Its explaining ability makes it suitable for medical experts. However, building accurate networks requires substantial domain knowledge.

Based on these alternatives, the attributes of AI-powered models are given by.

##### Predictive analysis

AI models leverage predictive algorithms to anticipate potential issues, such as the risk of falls, disease progression, or the likelihood of therapy success. This allows for proactive adjustments in treatment plans.

##### Automation of routine tasks

AI can automate repetitive and time-consuming tasks, such as generating progress reports, analyzing diagnostic data, or scheduling rehabilitation sessions, allowing healthcare professionals to focus on patient care.

##### Interactive and engaging interface

Many AI systems use virtual assistants, gamification, or augmented/virtual reality to make rehabilitation exercises more interactive and engaging for patients, improving compliance and motivation.

##### Real-time monitoring and feedback

AI enables real-time monitoring of patient activities and provides instant feedback to ensure exercises or therapies are performed correctly. This can help prevent injuries and improve outcomes.

The WVs of attributes are $\:\mathcal{w}=\left(0.21,\:0.23,\:0.36,\:0.20\right)$. To demonstrate the usefulness of the devised approach in decision-making, we must now use the suggested algorithm to choose the optimal option based on these facts.

###### Step 1

The information provided by experts in the form of CFNs is given in Table 3.

**Table 3:** Tamir’s form CF information.

	$\:{\mathcal{S}}_{\varvec{a}\varvec{t}\varvec{r}.-1}$	$\:{\mathcal{S}}_{\varvec{a}\varvec{t}\varvec{r}.-2}$	$\:{\mathcal{S}}_{\varvec{a}\varvec{t}\varvec{r}.-3}$	$\:{\mathcal{S}}_{\varvec{a}\varvec{t}\varvec{r}.-4}$
$\:{\mathcal{T}}_{\varvec{a}\varvec{l}\varvec{t}-1}$	$\:\left(0.\:25+\iota\:0.27\right)$	$\:\left(0.\:17+\iota\:0.37\right)$	$\:\left(0.\:77+\iota\:0.59\right)$	$\:\left(0.\:47+\iota\:0.21\right)$
$\:{\mathcal{T}}_{\varvec{a}\varvec{l}\varvec{t}-2}$	$\:\left(0.\:15+\iota\:0.14\right)$	$\:\left(0.\:18+\iota\:0.38\right)$	$\:\left(0.\:87+\iota\:0.69\right)$	$\:\left(0.\:37+\iota\:0.29\right)$
$\:{\mathcal{T}}_{\varvec{a}\varvec{l}\varvec{t}-3}$	$\:\left(0.\:22+\iota\:0.24\right)$	$\:\left(0.\:19+\iota\:0.31\right)$	$\:\left(0.\:67+\iota\:0.79\right)$	$\:\left(0.\:36+\iota\:0.28\right)$
$\:{\mathcal{T}}_{\varvec{a}\varvec{l}\varvec{t}-4}$	$\:\left(0.\:10+\iota\:0.23\right)$	$\:\left(0.\:30+\iota\:0.35\right)$	$\:\left(0.\:57+\iota\:0.51\right)$	$\:\left(0.\:34+\iota\:0.35\right)$
$\:{\mathcal{T}}_{\varvec{a}\varvec{l}\varvec{t}-5}$	$\:\left(0.\:11+\iota\:0.22\right)$	$\:\left(0.\:27+\iota\:0.39\right)$	$\:\left(0.\:47+\iota\:0.49\right)$	$\:\left(0.\:35+\iota\:0.11\right)$
$\:{\mathcal{T}}_{\varvec{a}\varvec{l}\varvec{t}-6}$	$\:\left(0.\:12+\iota\:0.21\right)$	$\:\left(0.\:38+\iota\:0.38\right)$	$\:\left(0.\:37+\iota\:0.39\right)$	$\:\left(0.\:17+\iota\:0.19\right)$
$\:{\mathcal{T}}_{\varvec{a}\varvec{l}\varvec{t}-7}$	$\:\left(0.\:13+\iota\:0.20\right)$	$\:\left(0.\:49+\iota\:0.83\right)$	$\:\left(0.\:27+\iota\:0.29\right)$	$\:\left(0.\:16+\iota\:0.18\right)$
$\:{\mathcal{T}}_{\varvec{a}\varvec{l}\varvec{t}-8}$	$\:\left(0.\:14+\iota\:0.19\right)$	$\:\left(0.\:50+\iota\:0.96\right)$	$\:\left(0.\:17+\iota\:0.28\right)$	$\:\left(0.\:14+\iota\:0.15\right)$
$\:{\mathcal{T}}_{\varvec{a}\varvec{l}\varvec{t}-9}$	$\:\left(0.\:15+\iota\:0.18\right)$	$\:\left(0.\:67+\iota\:0.99\right)$	$\:\left(0.\:10+\iota\:0.5\right)$	$\:\left(0.\:19+\iota\:0.10\right)$
$\:{\mathcal{T}}_{\varvec{a}\varvec{l}\varvec{t}-10}$	$\:\left(0.\:13+\iota\:0.12\right)$	$\:\left(0.\:11+\iota\:0.19\right)$	$\:\left(0.\:14+\iota\:0.16\right)$	$\:\left(0.\:18+\iota\:0.13\right)$

###### Stage 2

No need to normalize the information given in Table 3 because all the data is of benefit type.

###### Stage 3

The relative importance of the alternative is denoted and computed by using the formula of CFAAWA AO, that is.

$$\:{\mathcal{H}}_{\mathcal{j}}^{WS}=\left(1-{e}^{-{\left(\sum\:_{\mathcal{j}\,=1}^{\mathbbm{n}}{\mathcal{w}}_{\mathcal{j}}{\left(-\text{log}\left(1-{\mathcal{a}}_{\mathcal{j}}\right)\right)}^{\mathcal{r}}\right)}^{\frac{1}{\mathcal{r}}}}+\:\iota\:\:\left(1-{e}^{-{\left(\sum\:_{\mathcal{j}\,=1}^{\mathbbm{n}}{\mathcal{w}}_{\mathcal{j}}{\left(-\text{log}\left(1-{b}_{\mathcal{j}}\right)\right)}^{\mathcal{r}}\right)}^{\frac{1}{\mathcal{r}}}}\right)\right)$$

$$\:{\mathcal{H}}_{1}^{WS}=0.2809+\iota\:0.2029,\:{\mathcal{H}}_{2}^{WS}=0.3254+\iota\:0.2399,$$

$$\:{\mathcal{H}}_{3}^{WS}=0.2257+\iota\:0.2844,\:{\mathcal{H}}_{4}^{WS}=0.1920+\iota\:0.1941$$

$$\:{\mathcal{H}}_{5}^{WS}=0.1636+\iota\:0.1709,\:{\mathcal{H}}_{6}^{WS}=0.1374+\iota\:0.1519,$$

$$\:{\mathcal{H}}_{7}^{WS}=0.1343+\iota\:0.2351,\:{\mathcal{H}}_{8}^{WS}=0.1177+\iota\:0.3338$$

$$\:{\mathcal{H}}_{9}^{WS}=0.1481+\iota\:0.4487,\:{\mathcal{H}}_{10}^{WS}=0.0630+\iota\:0.0695$$

###### Step 4

Similarly, find out $\:{\mathcal{H}}_{\mathcal{j}}^{WP}$ by using the formula of CFAAWG AOs as

$$\:{\mathcal{H}}_{\mathcal{j}}^{WP}=\left({e}^{-{\left(\sum\:_{\mathcal{j}=1}^{\mathbbm{n}}{\mathcal{w}}_{\mathcal{j}}{\left(-\text{log}\left({\mathcal{a}}_{\mathcal{j}}\right)\right)}^{\mathcal{r}}\right)}^{\frac{1}{\mathcal{r}}}}+\:\iota\:\:\left({e}^{-{\left(\sum\:_{\mathcal{j}=1}^{\mathbbm{n}}{\mathcal{w}}_{\mathcal{j}}{\left(-\text{log}\left({b}_{\mathcal{j}}\right)\right)}^{\mathcal{r}}\right)}^{\frac{1}{\mathcal{r}}}}\right)\right)$$

$$\:{\mathcal{H}}_{1}^{WP}=0.6637+0.6461,\:{\mathcal{H}}_{2}^{WP}=0.6361+\iota\:0.6431,$$

$$\:{\mathcal{H}}_{3}^{WP}=0.6342+\iota\:0.6740,\:{\mathcal{H}}_{4}^{WP}=0.5994+\iota\:0.6470,\:$$

$$\:{\mathcal{H}}_{5}^{WP}=0.5820+\iota\:0.5854,{\mathcal{H}}_{6}^{WP}=0.5491+\iota\:0.5883\:$$

$$\:{\mathcal{H}}_{7}^{WP}=0.5372+\iota\:0.6017,\:{\mathcal{H}}_{8}^{WP}=0.4983+\iota\:0.5949,$$

$$\:{\mathcal{H}}_{9}^{WP}=0.4880+\iota\:0.6276,\:{\mathcal{H}}_{10}^{WP}=0.4219+\iota\:0.4391$$

###### Step 5

Finally, use the WASPAS approach.

$$\:{\mathcal{H}}_{\mathcal{j}}=\frac{{\mathcal{H}}_{\mathcal{j}}^{WS}+{\mathcal{H}}_{\mathcal{j}}^{WP}}{2}$$

And get the results

$$\:{\mathcal{H}}_{1}=0.1252+\iota\:0.1124,\:{\mathcal{H}}_{2}=,\:0.1240+\iota\:0.1157,$$

$$\:{\mathcal{H}}_{3}=0.1121+\iota\:0.1282,\:{\mathcal{H}}_{4}=0.1009+\iota\:0.1117,$$

$$\:{\mathcal{H}}_{5}=0.0943+\iota\:0.0958,\:{\mathcal{H}}_{6}==0.0852+\iota\:0.0944$$

$$\:{\mathcal{H}}_{7}=0.0826+\iota\:0.1060,\:{\mathcal{H}}_{8}=0.0739+\iota\:0.1161,$$

$$\:\:{\mathcal{H}}_{9}=0.0752+\iota\:0.1386,\:{\mathcal{H}}_{10}=0.0561+\iota\:0.0594\:$$

###### Step 6

Now use the formula of the score function to get the score values of the alternatives and then arrange these results to get the ranking order. Decide the best alternative based on the information of ranking order. The ranking results are given in Table 4.

**Table 4:** Score value and ranking results.

Score values	Ranking Result
$\:\varvec{S}\varvec{c}\varvec{r}.\:\left({\mathcal{H}}_{1}\right)=0.1188$	$$\begin{aligned}&\:{\mathcal{T}}_{alt-3}>{\mathcal{T}}_{alt-2}>{\mathcal{T}}_{alt-1}>\\&{\mathcal{T}}_{alt-9}>{\mathcal{T}}_{alt-4}>{\mathcal{T}}_{alt-8}>\\&{\mathcal{T}}_{alt-5}>{\mathcal{T}}_{alt-7}>{\mathcal{T}}_{alt-6}>{\mathcal{T}}_{alt-10}\end{aligned}$$
$$\:\varvec{S}\varvec{c}\varvec{r}.\:\left({\mathcal{H}}_{2}\right)=0.1199$$	
$$\:\varvec{S}\varvec{c}\varvec{r}.\:\left({\mathcal{H}}_{3}\right)=0.1202$$	
$$\:\varvec{S}\varvec{c}\varvec{r}.\:\left({\mathcal{H}}_{4}\right)=0.1063$$	
$$\:\varvec{S}\varvec{c}\varvec{r}.\:\left({\mathcal{H}}_{5}\right)=0.095078$$	
$$\:\varvec{S}\varvec{c}\varvec{r}.\:\left({\mathcal{H}}_{6}\right)=0.0898$$	
$$\:\varvec{S}\varvec{c}\varvec{r}.\:\left({\mathcal{H}}_{7}\right)=0.0943$$	
$$\:\varvec{S}\varvec{c}\varvec{r}.\:\left({\mathcal{H}}_{8}\right)=0.095087$$	
$$\:\varvec{S}\varvec{c}\varvec{r}.\:\left({\mathcal{H}}_{9}\right)=0.1069$$	
$$\:\varvec{S}\varvec{c}\varvec{r}.\:\left({\mathcal{H}}_{10}\right)=0.0578$$	

From the analysis of the results obtained from Table 3, we can conclude that$\:"{\mathcal{T}}_{\varvec{a}\varvec{l}\varvec{t}-3}"$ is the best alternative.

**Fig. 3:**
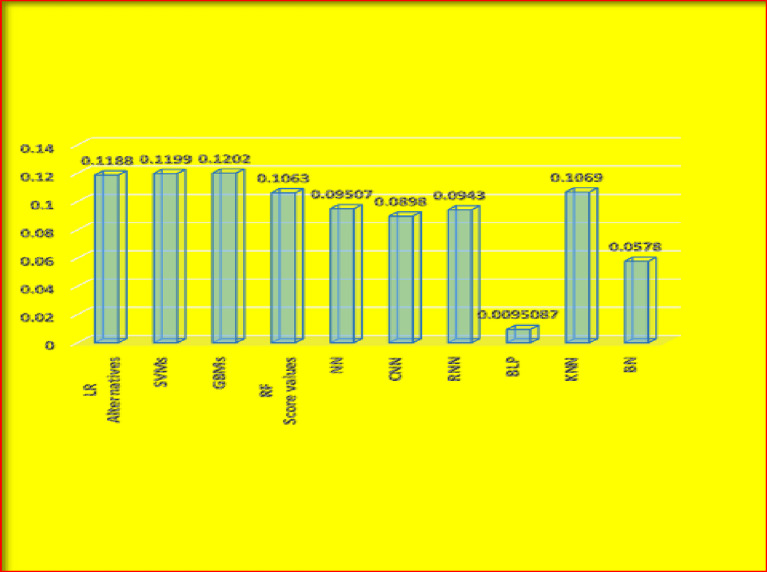
Graphical representation of results in Table 4.

The graphical representation of the results given in Table 4 is given in Fig. 3.

Figure 3 shows score values and ranking of the different alternatives. We can observe from the diagram that GBM is the best alternative. The score values for all these alternatives are given in the graph, and we can see that the highest value is 0.1202, which is against GBMs in the diagram. We can easily select the best alternative, and their ranking result can be managed according to their results.

## Comparative analysis

This part of the article is about the comparative analysis of the proposed approach. In this section, we show how this approach is more suitable and reliable than other existing notions. Here we have utilized the results by using the notion of CFAAWA and CFAAWG AOs. We have compared our work with different existing notions to deliver the advantages and reliability of the introduced work. For results, we have used the data from Table 3. The overall results are given in Table 5.

**Table 5:** Results of comparative analysis.

Methods	Score values	Ranking results
*Mardani et al*^34^. *Approach*	Fail to hold	No results
*Merigo et al*^35^. *Approach*	Fail to hold	No results
*Mardani et al.*^36^ *Approach*	*Fail to hold*	*No results*
*Turskis et al.* ^37^ *Approach*	*Fail to hold*	*No results*
*Pamucar et al.*^38^ *Approach*	*Fail to hold*	*No results*
*Hu et al.*^39^ *Approach*	*Fail to hold*	*No results*
*Bi et al.*^40^^[,41^ *Approach*	*Fail to hold*	*No results*
*CFAAWA AOs (Proposed)*	$\:Scr.\:\left({\mathcal{H}}_{1}\right)=0.2450$ $\:Scr.\:\left({\mathcal{H}}_{2}\right)=0.2827$ $\:Scr.\:\left({\mathcal{H}}_{3}\right)=0.2550$ $\:Scr.\:\left({\mathcal{H}}_{4}\right)=0.1930$ $\:Scr.\:\left({\mathcal{H}}_{5}\right)=0.1673$ $\:Scr.\:\left({\mathcal{H}}_{6}\right)=0.1447$ $\:Scr.\:\left({\mathcal{H}}_{7}\right)=0.1884$ $\:Scr.\:\left({\mathcal{H}}_{8}\right)=0.2258$ $\:Scr.\:\left({\mathcal{H}}_{9}\right)=0.2985$ $\:Scr.\:\left({\mathcal{H}}_{10}\right)=0.0663$	$$\begin{aligned}&\:{\mathcal{T}}_{\varvec{a}\varvec{l}\varvec{t}-9}>{\mathcal{T}}_{\varvec{a}\varvec{l}\varvec{t}-2}>{\mathcal{T}}_{\varvec{a}\varvec{l}\varvec{t}-3}>\\&{\mathcal{T}}_{\varvec{a}\varvec{l}\varvec{t}-1}>{\mathcal{T}}_{\varvec{a}\varvec{l}\varvec{t}-8}>{\mathcal{T}}_{\varvec{a}\varvec{l}\varvec{t}-4}>\\&{\mathcal{T}}_{\varvec{a}\varvec{l}\varvec{t}-7}>{\mathcal{T}}_{\varvec{a}\varvec{l}\varvec{t}-5}>{\mathcal{T}}_{\varvec{a}\varvec{l}\varvec{t}-6}>{\mathcal{T}}_{\varvec{a}\varvec{l}\varvec{t}-10}\end{aligned}$$
*CFAAWG AOs (Proposed)*	$\:Scr.\:\left({\mathcal{H}}_{1}\right)=0.6549$ $\:Scr.\:\left({\mathcal{H}}_{2}\right)=0.6396$ $\:Scr.\:\left({\mathcal{H}}_{3}\right)=0.6541$ $\:Scr.\:\left({\mathcal{H}}_{4}\right)=0.6232$ $\:Scr.\:\left({\mathcal{H}}_{5}\right)=0.5837$ $\:Scr.\:\left({\mathcal{H}}_{6}\right)=0.5687$ $\:Scr.\:\left({\mathcal{H}}_{7}\right)=0.5695$ $\:Scr.\:\left({\mathcal{H}}_{8}\right)=0.5467$ $\:Scr.\:\left({\mathcal{H}}_{9}\right)=0.5579$ $\:Scr.\:\left({\mathcal{H}}_{10}\right)=0.4305$	$$\begin{aligned}&\:{\mathcal{T}}_{\varvec{a}\varvec{l}\varvec{t}-1}>{\mathcal{T}}_{\varvec{a}\varvec{l}\varvec{t}-3}>{\mathcal{T}}_{\varvec{a}\varvec{l}\varvec{t}-2}>\\&{\mathcal{T}}_{\varvec{a}\varvec{l}\varvec{t}-4}>{\mathcal{T}}_{\varvec{a}\varvec{l}\varvec{t}-5}>{\mathcal{T}}_{\varvec{a}\varvec{l}\varvec{t}-7}>\\&{\mathcal{T}}_{\varvec{a}\varvec{l}\varvec{t}-6}>{\mathcal{T}}_{\varvec{a}\varvec{l}\varvec{t}-9}>{\mathcal{T}}_{\varvec{a}\varvec{l}\varvec{t}-8}>{\mathcal{T}}_{\varvec{a}\varvec{l}\varvec{t}-10}\end{aligned}$$

1. In the case of Mardani et al.^34^ and Merigo et al.^35^ approach, we can see that they have developed the decision-making method under the environment of fuzzy sets and AOs. Although the delivered application justifies the application in the fuzzy case and it generalizes the crisp set theory. But still, this approach is limited and has drawbacks. This approach can never discuss the two-dimensional information like complex fuzzy data as proposed in the introduced AOs. Then, these developed approaches can never discuss the AI-powered prediction models for tracking disability diseases. Hence, the defined approach can only play a vital role, and it can discuss the two-dimensional information effectively. So the introduced ideas are more reliable and supportive of human disability.
2. Also, in the case of the Mardani et al.^36^ approach, we can see that they have delivered the WASPAS approach based on fuzzy structure, but still, this WASPAS approach is limited due to the restriction of the fuzzy structure. This approach can never discuss the complex fuzzy data, and hence, in this way, it has the drawback of not discovering advanced data.
3. In the case of the Turskis et al.^37^ approach and the Pamucar et al.^38^ approach, we can observe that in the Turskis et al.^37^ approach, fuzzy AHP and WASPAS approaches have been utilized to solve the sustainable development of EU countries in terms of identifying the critical information systems. In the case of Pamucar et al.^38^ approach, they discussed the decision-making approach and utilized the fuzzy WASPAS technique for recovery center selection for end-of-life automotive lithium-ion batteries. But we can see that both approaches are based on the fuzzy WASPAS approach, and in this case, the loss of information is obvious. When decision-makers provide their assessment in the Cartesian form of CFS, then the existing notion fails, and, in this case, two-dimensional information can never be covered. The developed approach can solve this problem and provide more space for decision-makers to handle the two-dimensional information effectively.
4. The Hu et al.^39^ approach is based on a fuzzy rough set model. Although this approach is a generalization of a fuzzy set, and it can consider the upper and lower approximation in the data but still this approach can never help if the decision makers provide the assessment in Tamir’s CFSs. Only the delivered approach can help in the decision-making of the data in the Cartesian form of CFS. Hence, the introduced approach is superior to existing notions.
5. In the case of the Bi et al.^40,41^ approach, we can see that these approaches are based on Ramot’s CFSs. However, this approach is less applicable due to its structural restriction that the amplitude term must belong to [0, 1]. If the decision-makers provide the assessment in Tamir’s CFS like $\:0.8+\iota\:0.9$ then the amplitude term in polar form is $\:r=\sqrt{{0.9}^{2}+{0.8}^{2}}\notin\:[\left[0,\:1\right]$. Hence, in this case, only Tamir’s CFS helps. As the developed ideas are based on Tamir’s CFSs, they provide a flexible method and technique to handle disability-based problems.

More discussion on comparative analysis is given by.

If we compare our work with the Mardani et al.^34^ approach, we can observe that this approach is based on fuzzy aggregation operators, and these operators can only deal with fuzzy data, but the data of the developed approach is based on Tamir’s fuzzy information. We can notice that the existing notion can never discuss the complex information, while the developed approach can handle this information. Hence, we can say that the introduced approach is more dominant than the Mardani et al.^34^ approach. Merigo et al.^35^ produced the idea of fuzzy induced generalized aggregation operators. One drawback of this idea is that they can never discuss the complex structure, and the other is that they are free-form parameters. The introduced ideas are based on both of these characteristics. Parameters play a vital role in decision-making situations. The introduced ideas have these advantages to cover both of these characteristics. Mardani et al.^36^ developed the WASPAS approach, Turskis et al.^37^ WASPAS approach, and Pamucar et al.^38^ approach in the framework of fuzzy set theory and fuzzy set theory only discusses the membership grade, while the proposed ideas depend on the membership grade in the form of a complex number. The introduced ideas provide more space in terms of handling more advanced data. The two-dimensional data can never be covered by the approach delivered in^37,38^, while the developed WASPAS technique can cover these situations. Hu et al.^39^ approach is based on fuzzy rough structure, and this structure can categorize the data into upper and lower approximations, but still, these approaches can never discuss Tamir’s complex fuzzy information. The introduced approach can cover this information and assist in the decision-making scenarios. In these developed approaches, the chance of data loss decreases. Bi et al.‘s^40,41^ approaches consist of complex fuzzy average and geometric aggregation operators, but these approaches are based on the idea of Ramot’s notion of complex fuzzy set, which is less applicable as compared to Tamir’s complex fuzzy notion. The proposed idea is based on Tamir’s complex fuzzy set. In the case of Ramot’s approaches, we can notice that the value of membership grade belongs to unit discs in the complex plane while in case of Tamir’s complex fuzzy set this value belong to unit square in the complex pane and unit square in the complex plane provide more space as compared to unit disc. Hence, in all these ways, the introduced ideas are flexible and dominant over existing approaches.

**Fig. 4:**
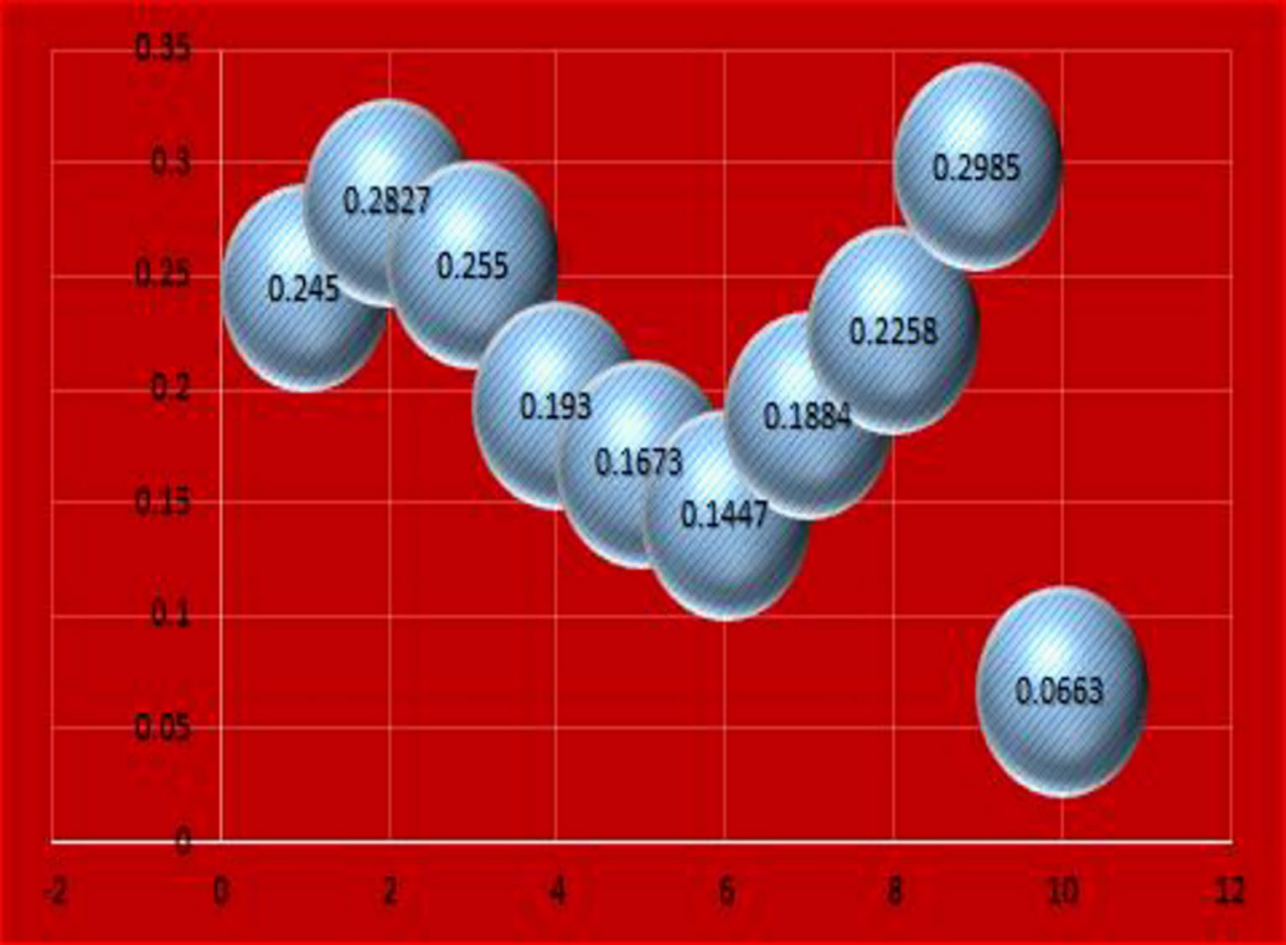
Graphical representation of results given in Table 5 for CFAAWA AOs.

**Fig. 5:**
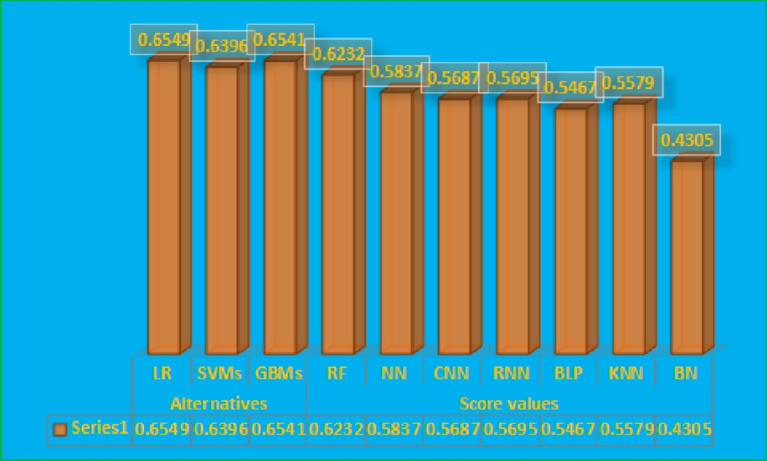
Graphical representation of results given in Table 5 for CFAAWG AOs.

Moreover, the graphical analysis of the results is given in Figs. 4 and 5.

From Figs. 4 and 5, we can analyze the aggregated results for CFAAWA AOs and CFAAWG AOs, respectively. We can see the score values for each alternative mentioned in both Figs. 4 and 5.

## Stability analysis based on parameter values

In this section, we have discussed the stability analysis of the established approach by using different values of parameters. We have analyzed whether the ranking result remains stable or not. If the ranking results remain stable, then we can ensure that the parameter does not affect the ranking values for this case, and if the ranking results change for both cases of CFAAWA AOs and CFAAWG AOs, then we can ensure that the effects of the parameter values on ranking results. For this purpose, we have utilized different values of the parameter, and the results are given in Table 6.

**Table 6:** Score values and ranking results for different parameter values.

Parameter Values	Score Values for CFAAWA AOs	Ranking Result for CFAAWA AOs	Score Values for CFAAWG AOs	Ranking Result for CFAAWG AOs
$\:\mathcal{r}=1$	$\:Scr.\:\left({\mathcal{H}}_{1}\right)=0.2450$ $\:Scr.\:\left({\mathcal{H}}_{2}\right)=0.2827$ $\:Scr.\:\left({\mathcal{H}}_{3}\right)=0.2550$ $\:Scr.\:\left({\mathcal{H}}_{4}\right)=0.1930$ $\:Scr.\:\left({\mathcal{H}}_{5}\right)=0.1673$ $\:Scr.\:\left({\mathcal{H}}_{6}\right)=0.1447$ $\:Scr.\:\left({\mathcal{H}}_{7}\right)=0.1884$ $\:Scr.\:\left({\mathcal{H}}_{8}\right)=0.2258$ $\:Scr.\:\left({\mathcal{H}}_{9}\right)=0.2985$ $\:Scr.\:\left({\mathcal{H}}_{10}\right)=0.0663$	$$\begin{aligned}&\:{\mathcal{T}}_{\varvec{a}\varvec{l}\varvec{t}-9}>{\mathcal{T}}_{\varvec{a}\varvec{l}\varvec{t}-2}>{\mathcal{T}}_{\varvec{a}\varvec{l}\varvec{t}-3}>\\&{\mathcal{T}}_{\varvec{a}\varvec{l}\varvec{t}-1}>{\mathcal{T}}_{\varvec{a}\varvec{l}\varvec{t}-8}>{\mathcal{T}}_{\varvec{a}\varvec{l}\varvec{t}-4}>\\&{\mathcal{T}}_{\varvec{a}\varvec{l}\varvec{t}-7}>{\mathcal{T}}_{\varvec{a}\varvec{l}\varvec{t}-5}>{\mathcal{T}}_{\varvec{a}\varvec{l}\varvec{t}-6}>{\mathcal{T}}_{\varvec{a}\varvec{l}\varvec{t}-10}\end{aligned}$$	$\:Scr.\:\left({\mathcal{H}}_{1}\right)=0.6549$ $\:Scr.\:\left({\mathcal{H}}_{2}\right)=0.6396$ $\:Scr.\:\left({\mathcal{H}}_{3}\right)=0.6541$ $\:Scr.\:\left({\mathcal{H}}_{4}\right)=0.6232$ $\:Scr.\:\left({\mathcal{H}}_{5}\right)=0.5837$ $\:Scr.\:\left({\mathcal{H}}_{6}\right)=0.5687$ $\:Scr.\:\left({\mathcal{H}}_{7}\right)=0.5695$ $\:Scr.\:\left({\mathcal{H}}_{8}\right)=0.5467$ $\:Scr.\:\left({\mathcal{H}}_{9}\right)=0.5579$ $\:Scr.\:\left({\mathcal{H}}_{10}\right)=0.4305$	$$\begin{aligned}&\:{\mathcal{T}}_{\varvec{a}\varvec{l}\varvec{t}-1}>{\mathcal{T}}_{\varvec{a}\varvec{l}\varvec{t}-3}>{\mathcal{T}}_{\varvec{a}\varvec{l}\varvec{t}-2}>\\&{\mathcal{T}}_{\varvec{a}\varvec{l}\varvec{t}-4}>{\mathcal{T}}_{\varvec{a}\varvec{l}\varvec{t}-5}>{\mathcal{T}}_{\varvec{a}\varvec{l}\varvec{t}-7}>\\&{\mathcal{T}}_{\varvec{a}\varvec{l}\varvec{t}-6}>{\mathcal{T}}_{\varvec{a}\varvec{l}\varvec{t}-9}>{\mathcal{T}}_{\varvec{a}\varvec{l}\varvec{t}-8}>{\mathcal{T}}_{\varvec{a}\varvec{l}\varvec{t}-10}\end{aligned}$$
$\:\mathcal{r}=2$	$\:Scr.\:\left({\mathcal{H}}_{1}\right)=0.2832$ $\:Scr.\:\left({\mathcal{H}}_{2}\right)=0.3493$ $\:Scr.\:\left({\mathcal{H}}_{3}\right)=0.3050$ $\:Scr.\:\left({\mathcal{H}}_{4}\right)=0.2115$ $\:Scr.\:\left({\mathcal{H}}_{5}\right)=0.1847$ $\:Scr.\:\left({\mathcal{H}}_{6}\right)=0.1549$ $\:Scr.\:\left({\mathcal{H}}_{7}\right)=0.2369$ $\:Scr.\:\left({\mathcal{H}}_{8}\right)=0.3192$ $\:Scr.\:\left({\mathcal{H}}_{9}\right)=0.4178$ $\:Scr.\:\left({\mathcal{H}}_{10}\right)=0.0674$	$$\begin{aligned}&\:{\mathcal{T}}_{\varvec{a}\varvec{l}\varvec{t}-9}>{\mathcal{T}}_{\varvec{a}\varvec{l}\varvec{t}-2}>{\mathcal{T}}_{\varvec{a}\varvec{l}\varvec{t}-8}>\\&{\mathcal{T}}_{\varvec{a}\varvec{l}\varvec{t}-3}>{\mathcal{T}}_{\varvec{a}\varvec{l}\varvec{t}-1}>{\mathcal{T}}_{\varvec{a}\varvec{l}\varvec{t}-7}>\\&{\mathcal{T}}_{\varvec{a}\varvec{l}\varvec{t}-4}>{\mathcal{T}}_{\varvec{a}\varvec{l}\varvec{t}-5}>{\mathcal{T}}_{\varvec{a}\varvec{l}\varvec{t}-6}>{\mathcal{T}}_{\varvec{a}\varvec{l}\varvec{t}-10}\end{aligned}$$	$\:Scr.\:\left({\mathcal{H}}_{1}\right)=0.6191$ $\:Scr.\:\left({\mathcal{H}}_{2}\right)=0.5869$ $\:Scr.\:\left({\mathcal{H}}_{3}\right)=0.6180$ $\:Scr.\:\left({\mathcal{H}}_{4}\right)=0.5972$ $\:Scr.\:\left({\mathcal{H}}_{5}\right)=0.5546$ $\:Scr.\:\left({\mathcal{H}}_{6}\right)=0.5535$ $\:Scr.\:\left({\mathcal{H}}_{7}\right)=0.5437$ $\:Scr.\:\left({\mathcal{H}}_{8}\right)=0.5165$ $\:Scr.\:\left({\mathcal{H}}_{9}\right)=0.5054$ $\:Scr.\:\left({\mathcal{H}}_{10}\right)=0.4292$	$$\begin{aligned}&\:{\mathcal{T}}_{\varvec{a}\varvec{l}\varvec{t}-1}>{\mathcal{T}}_{\varvec{a}\varvec{l}\varvec{t}-3}>{\mathcal{T}}_{\varvec{a}\varvec{l}\varvec{t}-4}>\\&{\mathcal{T}}_{\varvec{a}\varvec{l}\varvec{t}-2}>{\mathcal{T}}_{\varvec{a}\varvec{l}\varvec{t}-5}>{\mathcal{T}}_{\varvec{a}\varvec{l}\varvec{t}-6}>\\&{\mathcal{T}}_{\varvec{a}\varvec{l}\varvec{t}-7}>{\mathcal{T}}_{\varvec{a}\varvec{l}\varvec{t}-8}>{\mathcal{T}}_{\varvec{a}\varvec{l}\varvec{t}-9}>{\mathcal{T}}_{\varvec{a}\varvec{l}\varvec{t}-10}\end{aligned}$$
$\:\mathcal{r}=3$	$\:Scr.\:\left({\mathcal{H}}_{1}\right)=0.3094$ $\:Scr.\:\left({\mathcal{H}}_{2}\right)=0.3884$ $\:Scr.\:\left({\mathcal{H}}_{3}\right)=0.3390$ $\:Scr.\:\left({\mathcal{H}}_{4}\right)=0.2251$ $\:Scr.\:\left({\mathcal{H}}_{5}\right)=0.1965$ $\:Scr.\:\left({\mathcal{H}}_{6}\right)=0.1622$ $\:Scr.\:\left({\mathcal{H}}_{7}\right)=0.2750$ $\:Scr.\:\left({\mathcal{H}}_{8}\right)=0.3732$ $\:Scr.\:\left({\mathcal{H}}_{9}\right)=0.4816$ $\:Scr.\:\left({\mathcal{H}}_{10}\right)=0.0684$	$$\begin{aligned}&\:{\mathcal{T}}_{\varvec{a}\varvec{l}\varvec{t}-9}>{\mathcal{T}}_{\varvec{a}\varvec{l}\varvec{t}-2}>{\mathcal{T}}_{\varvec{a}\varvec{l}\varvec{t}-8}>\\&{\mathcal{T}}_{\varvec{a}\varvec{l}\varvec{t}-3}>{\mathcal{T}}_{\varvec{a}\varvec{l}\varvec{t}-1}>{\mathcal{T}}_{\varvec{a}\varvec{l}\varvec{t}-7}>\\&{\mathcal{T}}_{\varvec{a}\varvec{l}\varvec{t}-4}>{\mathcal{T}}_{\varvec{a}\varvec{l}\varvec{t}-5}>{\mathcal{T}}_{\varvec{a}\varvec{l}\varvec{t}-6}>{\mathcal{T}}_{\varvec{a}\varvec{l}\varvec{t}-10}\end{aligned}$$	$\:Scr.\:\left({\mathcal{H}}_{1}\right)=0.5949$ $\:Scr.\:\left({\mathcal{H}}_{2}\right)=0.5554$ $\:Scr.\:\left({\mathcal{H}}_{3}\right)=0.5965$ $\:Scr.\:\left({\mathcal{H}}_{4}\right)=0.5745$ $\:Scr.\:\left({\mathcal{H}}_{5}\right)=0.5279$ $\:Scr.\:\left({\mathcal{H}}_{6}\right)=0.5394$ $\:Scr.\:\left({\mathcal{H}}_{7}\right)=0.5282$ $\:Scr.\:\left({\mathcal{H}}_{8}\right)=0.5016$ $\:Scr.\:\left({\mathcal{H}}_{9}\right)=0.4766$ $\:Scr.\:\left({\mathcal{H}}_{10}\right)=0.4279$	$$\begin{aligned}&\:{\mathcal{T}}_{\varvec{a}\varvec{l}\varvec{t}-3}>{\mathcal{T}}_{\varvec{a}\varvec{l}\varvec{t}-1}>{\mathcal{T}}_{\varvec{a}\varvec{l}\varvec{t}-4}>\\&{\mathcal{T}}_{\varvec{a}\varvec{l}\varvec{t}-2}>{\mathcal{T}}_{\varvec{a}\varvec{l}\varvec{t}-6}>{\mathcal{T}}_{\varvec{a}\varvec{l}\varvec{t}-7}>\\&{\mathcal{T}}_{\varvec{a}\varvec{l}\varvec{t}-5}>{\mathcal{T}}_{\varvec{a}\varvec{l}\varvec{t}-8}>{\mathcal{T}}_{\varvec{a}\varvec{l}\varvec{t}-9}>{\mathcal{T}}_{\varvec{a}\varvec{l}\varvec{t}-10}\end{aligned}$$
$\:\mathcal{r}=4$	$\:Scr.\:\left({\mathcal{H}}_{1}\right)=0.3269$ $\:Scr.\:\left({\mathcal{H}}_{2}\right)=0.4119$ $\:Scr.\:\left({\mathcal{H}}_{3}\right)=0.3605$ $\:Scr.\:\left({\mathcal{H}}_{4}\right)=0.2354$ $\:Scr.\:\left({\mathcal{H}}_{5}\right)=0.2048$ $\:Scr.\:\left({\mathcal{H}}_{6}\right)=0.1672$ $\:Scr.\:\left({\mathcal{H}}_{7}\right)=0.2999$ $\:Scr.\:\left({\mathcal{H}}_{8}\right)=0.4045$ $\:Scr.\:\left({\mathcal{H}}_{9}\right)=0.5167$ $\:Scr.\:\left({\mathcal{H}}_{10}\right)=0.0695$	$$\begin{aligned}&\:{\mathcal{T}}_{\varvec{a}\varvec{l}\varvec{t}-9}>{\mathcal{T}}_{\varvec{a}\varvec{l}\varvec{t}-2}>{\mathcal{T}}_{\varvec{a}\varvec{l}\varvec{t}-8}>\\&{\mathcal{T}}_{\varvec{a}\varvec{l}\varvec{t}-3}>{\mathcal{T}}_{\varvec{a}\varvec{l}\varvec{t}-1}>{\mathcal{T}}_{\varvec{a}\varvec{l}\varvec{t}-7}>\\&{\mathcal{T}}_{\varvec{a}\varvec{l}\varvec{t}-4}>{\mathcal{T}}_{\varvec{a}\varvec{l}\varvec{t}-5}>{\mathcal{T}}_{\varvec{a}\varvec{l}\varvec{t}-6}>{\mathcal{T}}_{\varvec{a}\varvec{l}\varvec{t}-10}\end{aligned}$$	$\:Scr.\:\left({\mathcal{H}}_{1}\right)=0.5781$ $\:Scr.\:\left({\mathcal{H}}_{2}\right)=0.5345$ $\:Scr.\:\left({\mathcal{H}}_{3}\right)=0.5830$ $\:Scr.\:\left({\mathcal{H}}_{4}\right)=0.5560$ $\:Scr.\:\left({\mathcal{H}}_{5}\right)=0.5057$ $\:Scr.\:\left({\mathcal{H}}_{6}\right)=0.5272$ $\:Scr.\:\left({\mathcal{H}}_{7}\right)=0.5173$ $\:Scr.\:\left({\mathcal{H}}_{8}\right)=0.4923$ $\:Scr.\:\left({\mathcal{H}}_{9}\right)=0.4582$ $\:Scr.\:\left({\mathcal{H}}_{10}\right)=0.4266$	$$\begin{aligned}&\:{\mathcal{T}}_{\varvec{a}\varvec{l}\varvec{t}-3}>{\mathcal{T}}_{\varvec{a}\varvec{l}\varvec{t}-1}>{\mathcal{T}}_{\varvec{a}\varvec{l}\varvec{t}-4}>\\&{\mathcal{T}}_{\varvec{a}\varvec{l}\varvec{t}-2}>{\mathcal{T}}_{\varvec{a}\varvec{l}\varvec{t}-6}>{\mathcal{T}}_{\varvec{a}\varvec{l}\varvec{t}-7}>\\&{\mathcal{T}}_{\varvec{a}\varvec{l}\varvec{t}-5}>{\mathcal{T}}_{\varvec{a}\varvec{l}\varvec{t}-8}>{\mathcal{T}}_{\varvec{a}\varvec{l}\varvec{t}-9}>{\mathcal{T}}_{\varvec{a}\varvec{l}\varvec{t}-10}\end{aligned}$$
$\:\mathcal{r}=5$	$\:Scr.\:\left({\mathcal{H}}_{1}\right)=0.3390$ $\:Scr.\:\left({\mathcal{H}}_{2}\right)=0.4271$ $\:Scr.\:\left({\mathcal{H}}_{3}\right)=0.3747$ $\:Scr.\:\left({\mathcal{H}}_{4}\right)=0.2431$ $\:Scr.\:\left({\mathcal{H}}_{5}\right)=0.2109$ $\:Scr.\:\left({\mathcal{H}}_{6}\right)=0.1708$ $\:Scr.\:\left({\mathcal{H}}_{7}\right)=0.3168$ $\:Scr.\:\left({\mathcal{H}}_{8}\right)=0.4242$ $\:Scr.\:\left({\mathcal{H}}_{9}\right)=0.5382$ $\:Scr.\:\left({\mathcal{H}}_{10}\right)=0.0705$	$$\begin{aligned}&\:{\mathcal{T}}_{\varvec{a}\varvec{l}\varvec{t}-9}>{\mathcal{T}}_{\varvec{a}\varvec{l}\varvec{t}-2}>{\mathcal{T}}_{\varvec{a}\varvec{l}\varvec{t}-8}>\\&{\mathcal{T}}_{\varvec{a}\varvec{l}\varvec{t}-3}>{\mathcal{T}}_{\varvec{a}\varvec{l}\varvec{t}-1}>{\mathcal{T}}_{\varvec{a}\varvec{l}\varvec{t}-7}>\\&{\mathcal{T}}_{\varvec{a}\varvec{l}\varvec{t}-4}>{\mathcal{T}}_{\varvec{a}\varvec{l}\varvec{t}-5}>{\mathcal{T}}_{\varvec{a}\varvec{l}\varvec{t}-6}>{\mathcal{T}}_{\varvec{a}\varvec{l}\varvec{t}-10}\end{aligned}$$	$\:Scr.\:\left({\mathcal{H}}_{1}\right)=0.5659$ $\:Scr.\:\left({\mathcal{H}}_{2}\right)=0.5194$ $\:Scr.\:\left({\mathcal{H}}_{3}\right)=0.5737$ $\:Scr.\:\left({\mathcal{H}}_{4}\right)=0.5413$ $\:Scr.\:\left({\mathcal{H}}_{5}\right)=0.4880$ $\:Scr.\:\left({\mathcal{H}}_{6}\right)=0.5170$ $\:Scr.\:\left({\mathcal{H}}_{7}\right)=0.5092$ $\:Scr.\:\left({\mathcal{H}}_{8}\right)=0.4855$ $\:Scr.\:\left({\mathcal{H}}_{9}\right)=0.4454$ $\:Scr.\:\left({\mathcal{H}}_{10}\right)=0.4254$	$$\begin{aligned}&\:{\mathcal{T}}_{\varvec{a}\varvec{l}\varvec{t}-3}>{\mathcal{T}}_{\varvec{a}\varvec{l}\varvec{t}-1}>{\mathcal{T}}_{\varvec{a}\varvec{l}\varvec{t}-4}>\\&{\mathcal{T}}_{\varvec{a}\varvec{l}\varvec{t}-2}>{\mathcal{T}}_{\varvec{a}\varvec{l}\varvec{t}-6}>{\mathcal{T}}_{\varvec{a}\varvec{l}\varvec{t}-7}>\\&{\mathcal{T}}_{\varvec{a}\varvec{l}\varvec{t}-5}>{\mathcal{T}}_{\varvec{a}\varvec{l}\varvec{t}-8}>{\mathcal{T}}_{\varvec{a}\varvec{l}\varvec{t}-9}>{\mathcal{T}}_{\varvec{a}\varvec{l}\varvec{t}-10}\end{aligned}$$
$\:\mathcal{r}=6$	$\:Scr.\:\left({\mathcal{H}}_{1}\right)=0.3478$ $\:Scr.\:\left({\mathcal{H}}_{2}\right)=0.4377$ $\:Scr.\:\left({\mathcal{H}}_{3}\right)=0.3846$ $\:Scr.\:\left({\mathcal{H}}_{4}\right)=0.2491$ $\:Scr.\:\left({\mathcal{H}}_{5}\right)=0.2155$ $\:Scr.\:\left({\mathcal{H}}_{6}\right)=0.1734$ $\:Scr.\:\left({\mathcal{H}}_{7}\right)=0.3288$ $\:Scr.\:\left({\mathcal{H}}_{8}\right)=0.4376$ $\:Scr.\:\left({\mathcal{H}}_{9}\right)=0.5525$ $\:Scr.\:\left({\mathcal{H}}_{10}\right)=0.0715$	$$\begin{aligned}&\:{\mathcal{T}}_{\varvec{a}\varvec{l}\varvec{t}-9}>{\mathcal{T}}_{\varvec{a}\varvec{l}\varvec{t}-2}>{\mathcal{T}}_{\varvec{a}\varvec{l}\varvec{t}-8}>\\&{\mathcal{T}}_{\varvec{a}\varvec{l}\varvec{t}-3}>{\mathcal{T}}_{\varvec{a}\varvec{l}\varvec{t}-1}>{\mathcal{T}}_{\varvec{a}\varvec{l}\varvec{t}-7}>\\&{\mathcal{T}}_{\varvec{a}\varvec{l}\varvec{t}-4}>{\mathcal{T}}_{\varvec{a}\varvec{l}\varvec{t}-5}>{\mathcal{T}}_{\varvec{a}\varvec{l}\varvec{t}-6}>{\mathcal{T}}_{\varvec{a}\varvec{l}\varvec{t}-10}\end{aligned}$$	$\:Scr.\:\left({\mathcal{H}}_{1}\right)=0.5565$ $\:Scr.\:\left({\mathcal{H}}_{2}\right)=0.5081$ $\:Scr.\:\left({\mathcal{H}}_{3}\right)=0.5670$ $\:Scr.\:\left({\mathcal{H}}_{4}\right)=0.5296$ $\:Scr.\:\left({\mathcal{H}}_{5}\right)=0.4739$ $\:Scr.\:\left({\mathcal{H}}_{6}\right)=0.5087$ $\:Scr.\:\left({\mathcal{H}}_{7}\right)=0.5027$ $\:Scr.\:\left({\mathcal{H}}_{8}\right)=0.4804$ $\:Scr.\:\left({\mathcal{H}}_{9}\right)=0.4359$ $\:Scr.\:\left({\mathcal{H}}_{10}\right)=0.4242$	$$\begin{aligned}&\:{\mathcal{T}}_{\varvec{a}\varvec{l}\varvec{t}-3}>{\mathcal{T}}_{\varvec{a}\varvec{l}\varvec{t}-1}>{\mathcal{T}}_{\varvec{a}\varvec{l}\varvec{t}-4}>\\&{\mathcal{T}}_{\varvec{a}\varvec{l}\varvec{t}-2}>{\mathcal{T}}_{\varvec{a}\varvec{l}\varvec{t}-6}>{\mathcal{T}}_{\varvec{a}\varvec{l}\varvec{t}-7}>\\&{\mathcal{T}}_{\varvec{a}\varvec{l}\varvec{t}-8}>{\mathcal{T}}_{\varvec{a}\varvec{l}\varvec{t}-5}>{\mathcal{T}}_{\varvec{a}\varvec{l}\varvec{t}-9}>{\mathcal{T}}_{\varvec{a}\varvec{l}\varvec{t}-10}\end{aligned}$$
$\:\mathcal{r}=7$	$\:Scr.\:\left({\mathcal{H}}_{1}\right)=0.3543$ $\:Scr.\:\left({\mathcal{H}}_{2}\right)=0.4453$ $\:Scr.\:\left({\mathcal{H}}_{3}\right)=0.3918$ $\:Scr.\:\left({\mathcal{H}}_{4}\right)=0.2537$ $\:Scr.\:\left({\mathcal{H}}_{5}\right)=0.2192$ $\:Scr.\:\left({\mathcal{H}}_{6}\right)=0.1753$ $\:Scr.\:\left({\mathcal{H}}_{7}\right)=0.3377$ $\:Scr.\:\left({\mathcal{H}}_{8}\right)=0.4473$ $\:Scr.\:\left({\mathcal{H}}_{9}\right)=0.5627$ $\:Scr.\:\left({\mathcal{H}}_{10}\right)=0.0724$	$$\begin{aligned}&\:{\mathcal{T}}_{\varvec{a}\varvec{l}\varvec{t}-9}>{\mathcal{T}}_{\varvec{a}\varvec{l}\varvec{t}-8}>{\mathcal{T}}_{\varvec{a}\varvec{l}\varvec{t}-2}>\\&{\mathcal{T}}_{\varvec{a}\varvec{l}\varvec{t}-3}>{\mathcal{T}}_{\varvec{a}\varvec{l}\varvec{t}-1}>{\mathcal{T}}_{\varvec{a}\varvec{l}\varvec{t}-7}>\\&{\mathcal{T}}_{\varvec{a}\varvec{l}\varvec{t}-4}>{\mathcal{T}}_{\varvec{a}\varvec{l}\varvec{t}-5}>{\mathcal{T}}_{\varvec{a}\varvec{l}\varvec{t}-6}>{\mathcal{T}}_{\varvec{a}\varvec{l}\varvec{t}-10}\end{aligned}$$	$\:Scr.\:\left({\mathcal{H}}_{1}\right)=0.5491$ $\:Scr.\:\left({\mathcal{H}}_{2}\right)=0.4992$ $\:Scr.\:\left({\mathcal{H}}_{3}\right)=0.5618$ $\:Scr.\:\left({\mathcal{H}}_{4}\right)=0.5202$ $\:Scr.\:\left({\mathcal{H}}_{5}\right)=0.4628$ $\:Scr.\:\left({\mathcal{H}}_{6}\right)=0.5019$ $\:Scr.\:\left({\mathcal{H}}_{7}\right)=0.4974$ $\:Scr.\:\left({\mathcal{H}}_{8}\right)=0.4762$ $\:Scr.\:\left({\mathcal{H}}_{9}\right)=0.4285$ $\:Scr.\:\left({\mathcal{H}}_{10}\right)=0.4230$	$$\begin{aligned}&\:{\mathcal{T}}_{\varvec{a}\varvec{l}\varvec{t}-3}>{\mathcal{T}}_{\varvec{a}\varvec{l}\varvec{t}-1}>{\mathcal{T}}_{\varvec{a}\varvec{l}\varvec{t}-4}>\\&{\mathcal{T}}_{\varvec{a}\varvec{l}\varvec{t}-6}>{\mathcal{T}}_{\varvec{a}\varvec{l}\varvec{t}-2}>{\mathcal{T}}_{\varvec{a}\varvec{l}\varvec{t}-7}>\\&{\mathcal{T}}_{\varvec{a}\varvec{l}\varvec{t}-5}>{\mathcal{T}}_{\varvec{a}\varvec{l}\varvec{t}-8}>{\mathcal{T}}_{\varvec{a}\varvec{l}\varvec{t}-9}>{\mathcal{T}}_{\varvec{a}\varvec{l}\varvec{t}-10}\end{aligned}$$
$\:\mathcal{r}=8$	$\:Scr.\:\left({\mathcal{H}}_{1}\right)=0.3593$ $\:Scr.\:\left({\mathcal{H}}_{2}\right)=0.4512$ $\:Scr.\:\left({\mathcal{H}}_{3}\right)=0.3973$ $\:Scr.\:\left({\mathcal{H}}_{4}\right)=0.2575$ $\:Scr.\:\left({\mathcal{H}}_{5}\right)=0.2221$ $\:Scr.\:\left({\mathcal{H}}_{6}\right)=0.1768$ $\:Scr.\:\left({\mathcal{H}}_{7}\right)=0.3445$ $\:Scr.\:\left({\mathcal{H}}_{8}\right)=0.4546$ $\:Scr.\:\left({\mathcal{H}}_{9}\right)=0.5704$ $\:Scr.\:\left({\mathcal{H}}_{10}\right)=0.0732$	$$\begin{aligned}&\:{\mathcal{T}}_{\varvec{a}\varvec{l}\varvec{t}-9}>{\mathcal{T}}_{\varvec{a}\varvec{l}\varvec{t}-8}>{\mathcal{T}}_{\varvec{a}\varvec{l}\varvec{t}-2}>\\&{\mathcal{T}}_{\varvec{a}\varvec{l}\varvec{t}-3}>{\mathcal{T}}_{\varvec{a}\varvec{l}\varvec{t}-1}>{\mathcal{T}}_{\varvec{a}\varvec{l}\varvec{t}-7}>\\&{\mathcal{T}}_{\varvec{a}\varvec{l}\varvec{t}-4}>{\mathcal{T}}_{\varvec{a}\varvec{l}\varvec{t}-5}>{\mathcal{T}}_{\varvec{a}\varvec{l}\varvec{t}-6}>{\mathcal{T}}_{\varvec{a}\varvec{l}\varvec{t}-10}\end{aligned}$$	$\:Scr.\:\left({\mathcal{H}}_{1}\right)=0.5431$ $\:Scr.\:\left({\mathcal{H}}_{2}\right)=0.4922$ $\:Scr.\:\left({\mathcal{H}}_{3}\right)=0.5576$ $\:Scr.\:\left({\mathcal{H}}_{4}\right)=0.5126$ $\:Scr.\:\left({\mathcal{H}}_{5}\right)=0.4539$ $\:Scr.\:\left({\mathcal{H}}_{6}\right)=0.4963$ $\:Scr.\:\left({\mathcal{H}}_{7}\right)=0.4930$ $\:Scr.\:\left({\mathcal{H}}_{8}\right)=0.4728$ $\:Scr.\:\left({\mathcal{H}}_{9}\right)=0.4225$ $\:Scr.\:\left({\mathcal{H}}_{10}\right)=0.4219$	$$\begin{aligned}&\:{\mathcal{T}}_{\varvec{a}\varvec{l}\varvec{t}-3}>{\mathcal{T}}_{\varvec{a}\varvec{l}\varvec{t}-1}>{\mathcal{T}}_{\varvec{a}\varvec{l}\varvec{t}-4}>\\&{\mathcal{T}}_{\varvec{a}\varvec{l}\varvec{t}-6}>{\mathcal{T}}_{\varvec{a}\varvec{l}\varvec{t}-7}>{\mathcal{T}}_{\varvec{a}\varvec{l}\varvec{t}-2}>\\&{\mathcal{T}}_{\varvec{a}\varvec{l}\varvec{t}-8}>{\mathcal{T}}_{\varvec{a}\varvec{l}\varvec{t}-5}>{\mathcal{T}}_{\varvec{a}\varvec{l}\varvec{t}-9}>{\mathcal{T}}_{\varvec{a}\varvec{l}\varvec{t}-10}\end{aligned}$$
$\:\mathcal{r}=9$	$\:Scr.\:\left({\mathcal{H}}_{1}\right)=0.3633$ $\:Scr.\:\left({\mathcal{H}}_{2}\right)=0.4557$ $\:Scr.\:\left({\mathcal{H}}_{3}\right)=0.4016$ $\:Scr.\:\left({\mathcal{H}}_{4}\right)=0.2605$ $\:Scr.\:\left({\mathcal{H}}_{5}\right)=0.2246$ $\:Scr.\:\left({\mathcal{H}}_{6}\right)=0.1780$ $\:Scr.\:\left({\mathcal{H}}_{7}\right)=0.3499$ $\:Scr.\:\left({\mathcal{H}}_{8}\right)=0.4603$ $\:Scr.\:\left({\mathcal{H}}_{9}\right)=0.5764$ $\:Scr.\:\left({\mathcal{H}}_{10}\right)=0.0740$	$$\begin{aligned}&\:{\mathcal{T}}_{\varvec{a}\varvec{l}\varvec{t}-9}>{\mathcal{T}}_{\varvec{a}\varvec{l}\varvec{t}-8}>{\mathcal{T}}_{\varvec{a}\varvec{l}\varvec{t}-2}>\\&{\mathcal{T}}_{\varvec{a}\varvec{l}\varvec{t}-3}>{\mathcal{T}}_{\varvec{a}\varvec{l}\varvec{t}-1}>{\mathcal{T}}_{\varvec{a}\varvec{l}\varvec{t}-7}>\\&{\mathcal{T}}_{\varvec{a}\varvec{l}\varvec{t}-4}>{\mathcal{T}}_{\varvec{a}\varvec{l}\varvec{t}-5}>{\mathcal{T}}_{\varvec{a}\varvec{l}\varvec{t}-6}>{\mathcal{T}}_{\varvec{a}\varvec{l}\varvec{t}-10}\end{aligned}$$	$\:Scr.\:\left({\mathcal{H}}_{1}\right)=0.5381$ $\:Scr.\:\left({\mathcal{H}}_{2}\right)=0.4865$ $\:Scr.\:\left({\mathcal{H}}_{3}\right)=0.5542$ $\:Scr.\:\left({\mathcal{H}}_{4}\right)=0.5063$ $\:Scr.\:\left({\mathcal{H}}_{5}\right)=0.4467$ $\:Scr.\:\left({\mathcal{H}}_{6}\right)=0.4916$ $\:Scr.\:\left({\mathcal{H}}_{7}\right)=0.4892$ $\:Scr.\:\left({\mathcal{H}}_{8}\right)=0.4699$ $\:Scr.\:\left({\mathcal{H}}_{9}\right)=0.4175$ $\:Scr.\:\left({\mathcal{H}}_{10}\right)=0.4209$	$$\begin{aligned}&\:{\mathcal{T}}_{\varvec{a}\varvec{l}\varvec{t}-3}>{\mathcal{T}}_{\varvec{a}\varvec{l}\varvec{t}-1}>{\mathcal{T}}_{\varvec{a}\varvec{l}\varvec{t}-4}>\\&{\mathcal{T}}_{\varvec{a}\varvec{l}\varvec{t}-6}>{\mathcal{T}}_{\varvec{a}\varvec{l}\varvec{t}-7}>{\mathcal{T}}_{\varvec{a}\varvec{l}\varvec{t}-2}>\\&{\mathcal{T}}_{\varvec{a}\varvec{l}\varvec{t}-8}>{\mathcal{T}}_{\varvec{a}\varvec{l}\varvec{t}-5}>{\mathcal{T}}_{\varvec{a}\varvec{l}\varvec{t}-10}>{\mathcal{T}}_{\varvec{a}\varvec{l}\varvec{t}-9}\end{aligned}$$
$\:\mathcal{r}=10$	$\:Scr.\:\left({\mathcal{H}}_{1}\right)=0.3665$ $\:Scr.\:\left({\mathcal{H}}_{2}\right)=0.4594$ $\:Scr.\:\left({\mathcal{H}}_{3}\right)=0.4051$ $\:Scr.\:\left({\mathcal{H}}_{4}\right)=0.2629$ $\:Scr.\:\left({\mathcal{H}}_{5}\right)=0.2266$ $\:Scr.\:\left({\mathcal{H}}_{6}\right)=0.1790$ $\:Scr.\:\left({\mathcal{H}}_{7}\right)=0.3543$ $\:Scr.\:\left({\mathcal{H}}_{8}\right)=0.4649$ $\:Scr.\:\left({\mathcal{H}}_{9}\right)=0.5811$ $\:Scr.\:\left({\mathcal{H}}_{10}\right)=0.0747$	$$\begin{aligned}&\:{\mathcal{T}}_{\varvec{a}\varvec{l}\varvec{t}-9}>{\mathcal{T}}_{\varvec{a}\varvec{l}\varvec{t}-8}>{\mathcal{T}}_{\varvec{a}\varvec{l}\varvec{t}-2}>\\&{\mathcal{T}}_{\varvec{a}\varvec{l}\varvec{t}-3}>{\mathcal{T}}_{\varvec{a}\varvec{l}\varvec{t}-1}>{\mathcal{T}}_{\varvec{a}\varvec{l}\varvec{t}-7}>\\&{\mathcal{T}}_{\varvec{a}\varvec{l}\varvec{t}-4}>{\mathcal{T}}_{\varvec{a}\varvec{l}\varvec{t}-5}>{\mathcal{T}}_{\varvec{a}\varvec{l}\varvec{t}-6}>{\mathcal{T}}_{\varvec{a}\varvec{l}\varvec{t}-10}\end{aligned}$$	$\:Scr.\:\left({\mathcal{H}}_{1}\right)=0.5339$ $\:Scr.\:\left({\mathcal{H}}_{2}\right)=0.4819$ $\:Scr.\:\left({\mathcal{H}}_{3}\right)=0.5513$ $\:Scr.\:\left({\mathcal{H}}_{4}\right)=0.5010$ $\:Scr.\:\left({\mathcal{H}}_{5}\right)=0.4407$ $\:Scr.\:\left({\mathcal{H}}_{6}\right)=0.4877$ $\:Scr.\:\left({\mathcal{H}}_{7}\right)=0.4860$ $\:Scr.\:\left({\mathcal{H}}_{8}\right)=0.4673$ $\:Scr.\:\left({\mathcal{H}}_{9}\right)=0.4133$ $\:Scr.\:\left({\mathcal{H}}_{10}\right)=0.4198$	$$\begin{aligned}&\:{\mathcal{T}}_{\varvec{a}\varvec{l}\varvec{t}-3}>{\mathcal{T}}_{\varvec{a}\varvec{l}\varvec{t}-1}>{\mathcal{T}}_{\varvec{a}\varvec{l}\varvec{t}-4}>\\&{\mathcal{T}}_{\varvec{a}\varvec{l}\varvec{t}-6}>{\mathcal{T}}_{\varvec{a}\varvec{l}\varvec{t}-7}>{\mathcal{T}}_{\varvec{a}\varvec{l}\varvec{t}-2}>\\&{\mathcal{T}}_{\varvec{a}\varvec{l}\varvec{t}-8}>{\mathcal{T}}_{\varvec{a}\varvec{l}\varvec{t}-5}>{\mathcal{T}}_{\varvec{a}\varvec{l}\varvec{t}-10}>{\mathcal{T}}_{\varvec{a}\varvec{l}\varvec{t}-9}\end{aligned}$$

**Fig. 6:**
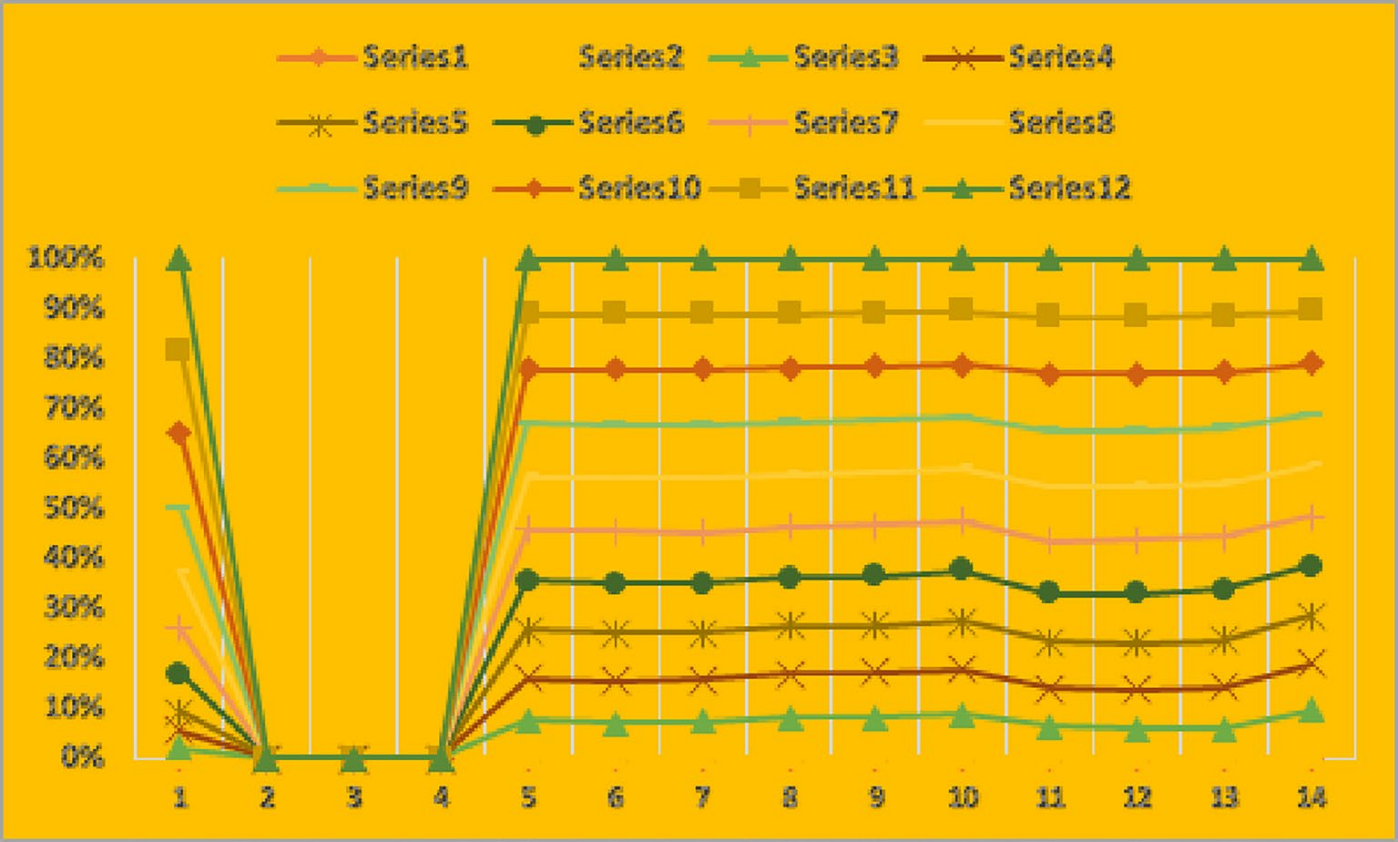
Graphical representation of data given in Table 6 for CFAAWA AOs.

- From the analysis of Table 6 above, we can observe that the ranking results slightly change for different values of the parameter, but there is no effect on the best alternative. The best alternative in all cases is the same, which shows the stability of the CFAAWA AOs.
- The graphical representation of the results obtained for different values of parameters in the case of CFAAWA AOs is given in Fig. 6.

Figure 6 represents the ranking result obtained by using the notion of CFAAWA AOs against different values of parameters ranging from^1,10^. We can see the stability of CFAAWA AOs through the ranking results.

**Fig. 7:**
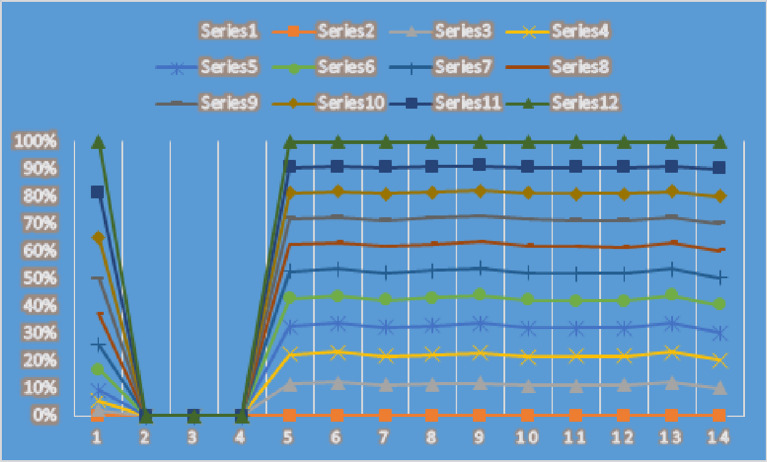
Graphical representation of data given in Table 6 for CFAAWG AOs.

- Also, in the case of utilizing the notion of CFAAWG AOs, we can observe from Table 5 that when 
- $\:\mathcal{r}=1\:\text{a}\text{n}\text{d}\:2,$ then in these cases, the best alternative is $\:{\mathcal{T}}_{alt-1}$ and when we increase the value of$\:\mathcal{r}=3,\:4,\:5,\:6,\:7,\:8,\:9,\:10,$ then the best alternative in this case is $\:{\mathcal{T}}_{alt-3}$. Hence this case, we can say that the CFAAG AOs remain unstable in^1^^,3^ and these AOS become stable in^3,10^ as discussed here in Table 6. Hence, in this case, we can say that the role of the parameter is effective in ranking the alternative results, and we can see different ranking results for different values of the parameter.
- Moreover, the geometrical representation of the data for CFAAWG AOs in Table 6 is given in Fig. 7.

Figure 7 shows the ranking result for CFAAWG AOs for different values of parameters. The CFAAG AOs are not stable in the interval [1, 3), and then they become stable in^3,10^. This behavior can be seen from the score values and ranking order of the different alternatives for different values of the parameter.

The overall summary of the sensitivity analysis is given by.

To ensure the robustness of the new Tamir’s Complex Fuzzy Aczel-Alsina WASPAS method, an extensive sensitivity analysis is performed. The main goal is to analyze the stability of the ranking outcomes when changing the distribution of the parameter. We changed the parameter values systematically, while others and we monitored their influence on the final ranking of the AI-based predictive models. The evaluation showed that the highest-ranked alternative was unchanged with a very broad range of parameters, showing extreme resilience and robustness of the presented method. Small ranking changes occurred only when very large parameter values were used, which proves that the choice-making procedure is not very sensitive to subjective parameters. This result substantiates the flexibility and stability of our approach in actual medical decision-making situations, where expert judgments and data imprecision can be dissimilar.

### Discussion of the key findings

The proposed Tamir’s Complex Fuzzy Aczel-Alsina WASPAS solution well addressed the problem of ranking AI-based prediction models for tracking disability development under uncertain and imprecise environments. The key findings are as follows:

1. Among the ranked AI models, the WASPAS approach ranked as the top one consistently since it performed excellently over multiple-choice parameters like accuracy, interpretability, and robustness.
2. The proposed theory can capture the uncertainty by using the idea of Tamir’s complex fuzzy set and Aczel-Alsina aggregation operators. The proposed WASPAS technique can handle the uncertain and ambiguous information in medical decision-making situations effectively as compared to other prevailing theories due to the parameter structure of Aczel-Alsina aggregation operators.
3. The result of the sensitivity analysis shows the stability of the delivered approach, and when we change the parameter values, then we can notice there are slight changes in ranking results and but they become stable when parameter values increase for $\:\mathcal{r}=3,\:4,\:5,\:6,\:7,\:8,\:9,\:10.$
4. This technique provides decision support systems and proves that these ideas can be used in medical files where the data is uncertain and ambiguous.
5. These findings validate the effectiveness of the proposed hybrid model in medical AI evaluation contexts and open new possibilities for decision-making under complex uncertainty.

## Conclusion

The study in this paper introduced a new hybrid multi-criteria decision-making method, Tamir’s Complex Fuzzy Aczel-Alsina WASPAS approach, to categorize and evaluate AI-based predictive models used in monitoring disability condition development. The innovative method sufficiently addressed the concerns of uncertainty, imprecision, and interdependency typically present in medical information through the integration of complex fuzzy logic’s expressiveness, Aczel-Alsina aggregation operator versatility, and WASPAS method’s two-stage robustness. By thorough analysis and sensitivity assessment, the model exhibited very high reliability, strength, and interpretability in ranking multiple AI models. The ranking stability across different weight distributions and comparative outperformance over conventional MCDM methods affirm the efficacy of the proposed model. The primary findings establish that the method can facilitate well-informed decisions within healthcare by identifying the best-fit AI models for early detection and ongoing tracking of disability diseases.

### Need, limitation, and impact of the proposed study

It has become increasingly necessary to provide intelligent decision-support systems that are able to help with the early detection and monitoring of disability conditions. Although there have been recent developments in AI-based prediction models, their classification and selection under uncertain, imprecise, and multidimensional medical data is still an open problem. This necessitates strong multi-criteria decision-making (MCDM) tools that are able to deal with such complexity.

Yet, current MCDM methods are not optimal in addressing the dual character of uncertainty (fuzziness and hesitation), interdependent criteria, and interpretability of outcomes in medicine. Our Tamir’s Complex Fuzzy Aczel-Alsina WASPAS scheme fills these shortcomings by presenting a new aggregation technique that integrates the expressiveness of complex fuzzy logic with Aczel-Alsina operators’ computational adaptability and the precision of WASPAS ranking.

Its significance is in its potential to enhance model evaluation transparency, improve decision-making accuracy for doctors, and outline a generalized framework for implementing in other uncertain settings outside disability prediction. It also advances the field toward uncertainty-aware AI frameworks that can sustain important applications in personalized medicine and public health policy.

The limitation of the proposed approach is obvious. As the developed approach is based on CF data, it can only discover the membership grade, but when data contains the non-membership grade or Abstinence grade, then the introduced approach fails to hold.

### Future study

In the future, the proposed approach can be generalized into rough set theory and soft set theory as developed in^42,43^. We can define the confidence level-based decision making as discussed in^44^ and other techniques like the MEREC-VIKOR method^45^ based on the developed approach.

## Data Availability

All data generated or analyzed during this study are included in this published article.
